# Cervical ripening at home or in-hospital—prospective cohort study and process evaluation (CHOICE) study: a protocol

**DOI:** 10.1136/bmjopen-2021-050452

**Published:** 2021-05-04

**Authors:** Sarah Jane Stock, Amarnath Bhide, Heather Richardson, Mairead Black, Cassandra Yuill, Mairi Harkness, Maggie Reid, Fiona Wee, Helen Cheyne, Christine McCourt, Dikshyanta Rana, Kathleen Anne Boyd, Julia Sanders, Neelam Heera, Jane Huddleston, Fiona Denison, Dharmintra Pasupathy, Neena Modi, Gordon Smith, John Norrie

**Affiliations:** 1 Centre for Medical Informatics, Usher Institute, University of Edinburgh, Edinburgh, UK; 2 Obstetrics and Gynaecology, St George’s University Hospitals Trust, London, UK; 3 Women and Children’s Health, NHS Lothian University Hospitals Division, Edinburgh, UK; 4 Institute of Applied Health Sciences, University of Aberdeen, Aberdeen, UK; 5 Centre for Maternal & Child Health Research, School of Health Sciences, City University of London, London, UK; 6 Nursing, Midwifery and Allied Health Professions Research Unit (NMAHP), University of Stirling, Stirling, UK; 7 Edinburgh Clinical Trials Unit (ECTU) Usher Institute, University of Edinburgh, Edinburgh, UK; 8 Institute of Health and Well Being, University of Glasgow, Glasgow, UK; 9 School of Healthcare Sciences, Cardiff University, Cardiff, UK; 10 Patient and Public Involvement Representative, Edinburgh, UK; 11 MRC Centre for Reproductive Health, The University of Edinburgh, Edinburgh, UK; 12 Faculty of Medicine and Health, The University of Sydney, Sydney, New South Wales, Australia; 13 Department of Medicine, Imperial College London, London, UK; 14 Department of Obstetrics and Gynaecology, University of Cambridge, Cambridge, UK

**Keywords:** maternal medicine, fetal medicine, obstetrics

## Abstract

**Introduction:**

The aim of the cervical ripening at home or in-hospital—prospective cohort study and process evaluation (CHOICE) study is to compare home versus in-hospital cervical ripening to determine whether home cervical ripening is safe (for the primary outcome of neonatal unit (NNU) admission), acceptable to women and cost-effective from the perspective of both women and the National Health Service (NHS).

**Methods and analysis:**

We will perform a prospective multicentre observational cohort study with an internal pilot phase. We will obtain data from electronic health records from at least 14 maternity units offering only in-hospital cervical ripening and 12 offering dinoprostone home cervical ripening. We will also conduct a cost-effectiveness analysis and a mixed methods study to evaluate processes and women/partner experiences. Our primary sample size is 8533 women with singleton pregnancies undergoing induction of labour (IOL) at 39+0 weeks’ gestation or more. To achieve this and contextualise our findings, we will collect data relating to a cohort of approximately 41 000 women undergoing IOL after 37 weeks. We will use mixed effects logistic regression for the non-inferiority comparison of NNU admission and propensity score matched adjustment to control for treatment indication bias. The economic analysis will be undertaken from the perspective of the NHS and Personal Social Services (PSS) and the pregnant woman. It will include a within-study cost-effectiveness analysis and a lifetime cost–utility analysis to account for any long-term impacts of the cervical ripening strategies. Outcomes will be reported as incremental cost per NNU admission avoided and incremental cost per quality adjusted life year gained.

**Research ethics approval and dissemination:**

CHOICE has been funded and approved by the National Institute of Healthcare Research Health Technology and Assessment, and the results will be disseminated via publication in peer-reviewed journals.

**Trial registration number:**

ISRCTN32652461.

**Strengths and limitations of this study**

- This is a large study to evaluate the safety of at-home cervical ripening.
- We will set up a platform for data collection using electronic health records to enable future research into rare safety outcomes.
- The study includes assessment of women’s views and experiences using validated questionnaires as well as qualitative methods.
- Observational design of the study makes it vulnerable to residual confounding.

## Introduction

Induction of labour (IOL) is the most common obstetric intervention offered to women when risks of continuing the pregnancy are thought to outweigh risks of birth. Increases in IOL rates over the past 10 years mean that now 29.6% of all pregnant women in the UK have their labour induced.^1^ IOL at term, when compared with expectant management of pregnancy, reduces caesarean birth, maternal hypertensive disease and complications,^2^ as well as being associated with a reduction in perinatal mortality.^3 4^ The demands on maternity services are increasing to accommodate increasing rates of IOL.^5^ Although IOL (compared with expectant management) reduces overall hospital stay, it increases the time on labour and delivery wards,^2^ having a major impact on resources, staffing and negatively impacts on women’s experience of labour.^6–8^

Cervical ripening is a key component of IOL.^9^ It may initiate labour, but is often followed by artificial rupture of membranes±intravenous oxytocin infusion. National Institute for Health and Care Excellence (NICE) guidance^10^ recommends pre-induction cervical ripening in all women having IOL unless there is a contraindication.

Traditionally, cervical ripening has been performed entirely in-hospital, to allow monitoring of maternal/fetal well-being and early recognition of complications.^11^ However, an increasing number of UK maternity units offers outpatient (home) cervical ripening. As the rate of IOL is increasing, home cervical ripening may provide opportunities to reduce the burden on the NHS, for example, by reducing hospital stay during IOL. However, the safety and acceptability of home cervical ripening have not been fully evaluated. NICE^10^ identified the need to assess the safety, efficacy and clinical and cost-effectiveness of outpatient and inpatient IOL in the UK setting, taking into account women’s views. A recent Cochrane review found insufficient evidence to draw conclusions on the efficacy, safety and cost-effectiveness of home IOL and indicated that large prospective cohort studies would be needed.^12^ Maternity service users have identified IOL as an important research topic,^13^ and women have reported specific negative experiences such as increased pain and anxiety and lack of support which may be alleviated by home cervical ripening.^8^ Potential NHS cost savings of home cervical ripening could be offset by increased costs of any additional morbidity resulting from home cervical ripening, costs to parents may be increased and acceptability of home cervical ripening is unknown. Health services need to balance the full resource impact of IOL with the need to provide safe and acceptable care.

In the cervical ripening at home or in-hospital—prospective cohort study and process evaluation (CHOICE) study, we will perform an observational cohort study with a cost-effectiveness analysis and process evaluation to address the question, ‘Is it safe, effective, cost-effective and acceptable to women to carry out home cervical ripening during IOL?’. These analyses will provide information to help women and their caregivers make informed decisions around how to have IOL.

Our main aim is to compare the setting of cervical ripening at home versus in-hospital. As the NICE recommended agent for cervical ripening is vaginal prostaglandin, our primary comparison will be home dinoprostone versus in-hospital dinoprostone. In order to future-proof the study, we will include a secondary comparison: home cervical ripening with balloon catheter versus home cervical ripening with dinoprostone. By including two different methods of home cervical ripening within our study, we will provide initial comparative evidence on these two methods of home labour induction.

## Methods and analysis

### CHOICE: a prospective cohort study

#### Study design and setting

We will carry out a prospective multicentre cohort study using de-identified clinical data from electronic hospital records. The primary outcome will be non-inferiority of a neonatal unit (NNU)/special care admission for 48 hours or longer, initiated within 48 hours of birth.

The study will be performed in at least 26 UK obstetric units, 14 of which offer exclusively in-hospital cervical ripening and 12 offer dinoprostone cervical ripening both in-hospital and at home.

Participating maternity units will be purposively selected to represent the diverse range of maternity service settings in the UK, and include urban tertiary referral units, mid-sized urban district general hospitals and small, more isolated, rural units.

#### Data sources

Data will be collected directly from electronic maternity (and neonatal) records for participants who had babies admitted to an NNU. These data are recorded by clinical staff (midwives, doctors and neonatal nurses) during the course of antenatal, intrapartum and postpartum care. Existing data fields, supplemented by new bespoke, data entry fields enabled in the maternity dataset at participating sites will be used. Unless women opt out of secondary data use (from similar studies, we estimate <1% will opt out), de-identified data will be transferred from participating sites to a secure University of Edinburgh server for analysis.

No personal data will be collected. Potentially identifiable data, such as the date and time of birth, date of events such as commencing cervical ripening and hospital discharge, will be converted into gestation at birth (weeks+days); and antenatal and postnatal events into ‘t–x’ and ‘t+x’ hours and days, respectively.

#### Population, inclusion and exclusion criteria

We will initially apply broad inclusion criteria and collect data from all women having IOL at 37+0 weeks’ gestation or more to create a cohort for analyses. Women who have opted out of data provision will be excluded.

We will then apply more stringent inclusion and exclusion criteria at the analysis stage for a suite of nested analyses. In our primary analysis, we will create a cohort of women with ‘uncomplicated’ (ie, those with no identified risk factors for adverse maternal or perinatal outcomes defined below) pregnancies in whom there was no contraindication to home cervical ripening, who had singleton pregnancies with IOL at 39 weeks’ gestation or more. This group will include women having IOL for post-dates, but also women having IOL because of maternal or clinician preference, IOL for maternal age and IOL for discomfort or social indications. Exclusion criteria will consist of grand multiparity (six or more previous births), previous caesarean section, antepartum stillbirth (before cervical ripening initiated), class III obesity at booking (body mass index (BMI): 40 kg/m^2^ or more), prelabour rupture of membranes (ROM) documented as primary or other indication for IOL (prolonged ROM, spontaneous ROM and suspected spontaneous ROM), maternal or fetal condition that would or could preclude home cervical ripening documented as primary or other indication for IOL (maternal conditions: proteinuria; hypertension; antepartum haemorrhage; diabetes; obstetric cholestasis; obstetric history; pre-eclampsia; pregnancy induced hypertension (PIH)/pre-eclampsia (PET) (not defined); PIH; PET; thrombophilia; fetal conditions: oligohydramnios; reduced liquor volume; macrosomia; intrauterine growth restriction; static growth; congenital fetal anomaly; polyhydramnios; abnormal cardiotocograph (CTG)/Doppler; breech; reduced fetal movements and termination of pregnancy for fetal anomaly).

We will explore the potential for additional analyses, which may include IOL for other indications (eg, reduced fetal movements) or in other populations (eg, multiple pregnancies and women with a previous caesarean birth). However, in general, home cervical ripening is only offered to ‘low risk’ women, so we anticipate that numbers of higher risk women having home IOL may not be high enough for meaningful analyses.

#### Exposure and outcomes

Our primary aim is to compare home versus in-hospital cervical ripening. We will collect data at individual level. The exposure group will be women who, at the start of the cervical ripening process, plan to have home cervical ripening. The comparator group will be women who planned to have in-hospital cervical ripening from maternity units not offering home cervical ripening. This will minimise potential bias arising from the fact that, in maternity units which offer both home and in-hospital cervical ripening, the risk of complications in the babies of women having home cervical ripening (lower risk pregnancies) is inherently different to that of babies of women having in-hospital cervical ripening (higher risk pregnancies).

As the NICE recommended agent for cervical ripening is vaginal prostaglandin, our primary comparison will be home dinoprostone versus in-hospital dinoprostone. Dinoprostone is now most commonly administered as 10-mg slow-release pessary (Propess, Ferring), which stays in place for 24 hours. We will use this formulation in our primary comparison.

We will include a secondary exploratory comparison—home cervical ripening with balloon catheter (exposure) versus home cervical ripening with dinoprostone (comparator), to explore if there are any indications of different safety profiles of these two methods of home cervical ripening.

Our proposed primary outcome will be admission to an NNU/special care baby unit for 48 hours or longer, initiated within 48 hours of birth. NNU admission is a marker of neonatal morbidity and is the leading core outcome defined for studies of IOL.^14^ Any increase in NNU admission of term babies is undesirable due to the separation of mother and baby. However, NNU admission rates are highly variable between maternity units and are likely to depend on local policies and culture. We, therefore, plan to use a primary outcome, which represents more severe neonatal morbidity (admission to a NNU within 48 hours of birth for 48 hours or longer), which is less likely to be influenced by site-specific factors. We may re-define the parameters of NNU admission used in the primary outcome after analysis of pilot data (see section ‘Pilot phase’).

We have prespecified a number of secondary outcomes to assess the safety of home cervical ripening with respect to neonatal and maternal morbidity, listed in table 1. These include outcomes from a core outcome set for studies of IOL.^14^ We will also include secondary outcomes relating to the effectiveness of home cervical ripening and to explore whether the setting of cervical ripening influences subsequent labour and birth. Mother and baby outcomes were suggested by our lay consultation as important to include. We will use birth weight, birth weight centile, small for gestational age and large for gestational age as parameters to check the validity of our matching procedures in analyses. Birth weight is an objective outcome that may represent pregnancy complications, but extremely unlikely to be affected by the setting of cervical ripening. Comparison of birth weights between groups should provide reassurance that we have minimised systemic bias in our analyses.

**Table 1:** Secondary outcomes

Safety outcomes	**Baby** Any neonatal unit (NNU) admission (any level of care) Neonatal intensive care unit (NICU) admission Duration of NNU stay Duration of NICU stay Apgarscore <7 at 5 min Apgar score <4 at 5 min Arterial cord blood pH <7.1 Arterial cord base excess >12 mmol/L Neonatal seizures Hypoxic ischaemic encephalopathy (as recorded by care givers) Level 2 or level 3 hypoxic ischaemic encephalopathy (as recorded by care givers) Meconium aspiration syndrome Mechanical ventilation Intracranial haemorrhage Stillbirth after admission/first attendance for induction of labour (excluding deaths from congenital anomalies) Early neonatal death up to 7 days after birth (day 0–6; excluding deaths from congenital anomalies) Treatment for neonatal sepsis (defined as positive blood, cerebral spinal fluid, or urine culture or cardiovascular collapse or X-ray confirming infection) (exploratory outcome) Treatment in NNU for neonatal infection (defined as antibiotic treatment and temperature ≥37.5°C or <35.5°C) (exploratory outcome) Treatment for neonatal jaundice (defined as peak total bilirubin of at least 15 mg or the use of phototherapy) (exploratory outcome)	**Maternal** Intensive care unit transfer High dependency level care Hyperstimulation or tachysystole (as defined by care givers) Hyperstimulation or tachysystole causing cardiotocograph (CTG) abnormality (as defined by care givers) Umbilical cord prolapse Birth outwith hospital Postpartum haemorrhage 1000 mL or more Maternal fever 38°C or more after commencing cervical ripening (exploratory outcome)
Effectiveness outcomes	Time from first cervical ripening agent to admission to labour ward/birth unit Time from first cervical ripening agent to birth More than one cervical ripening agent used Duration of antenatal hospital stay for cervical ripening Duration of labour ward admission until birth Duration postnatal hospital stay (mother) Total hospital stay Hours spent at home Oxytocin use Mode of birth Birth in obstetric unit Birth in alongside midwifery unit (if available at that site)	
Mother baby outcomes	Breastfeeding at discharge from maternity care Skin to skin at birth	
Cost effectiveness	**Primary health economic outcomes** Incremental cost per neonatal admissions avoided (home vs in-hospital) Incremental quality adjusted life year (QALYs) (home vs in-hospital) **Other (exploratory) economic outcomes** Incremental cost per hour prevented from hospital admission to delivery/birth Incremental cost per neonatal admission avoided (home balloon catheter vs home dinoprostone) Incremental cost per QALY (home balloon catheter vs home dinoprostone) Incremental cost per hour prevented from hospital admission to delivery/birth (home balloon catheter vs home dinoprostone)	
Outcomes to check comparability of groups/matching	Birth weight Birth weight centile Small for gestational age (<10th centile for gestational age) Large for gestational age (>90th centile for gestational age)	
Qualitative CHOICE process evaluation outcomes	**Primary qualitative outcome** Sense of control (agentry) in labour **Secondary qualitative outcomes** Women’s satisfaction with induction of labour care Women’s postnatal psychological well-being Women’s overall evaluation of their labour and birth experience (qualitative analysis) Costs incurred by the woman and family	

#### Statistical analysis

All analyses will be fully specified in a comprehensive Statistical Analysis Plan and agreed by the Steering Committee. Analyses will be carried out in accordance with relevant guidance, including Reporting of studies Conducted using Observational Routinely-collected Data (RECORD)^15^ and Strengthening the Reporting of Observational Studies in Epidemiology (STROBE).^16^

We will include at least 14 maternity units offering only in-hospital cervical ripening and 12 offering dinoprostone home cervical ripening (~95 000 deliveries per annum). We will invite additional maternity units to opt in to data provision, to allow contingency in case of ‘cross overs’ due to sites changing their IOL protocols during the study period.

We considered a superiority design for CHOICE, but decided against it because (a) safety is a key concern to both clinicians and women, and was specified as the important outcome in the commissioning brief; (b) it is not plausible to hypothesise that home cervical ripening (intervention) is safer than in-hospital cervical ripening (comparator—the standard of care) and (c) it is not ethical to use a superiority design to test an intervention, which may be worse (in terms of safety) than the established standard. Therefore, a non-inferiority design was chosen with a non-inferiority margin of 4% (deemed as likely to be an important difference on consultation with women and clinicians) for the primary outcome of NNU admission.

Establishing the appropriate non-inferiority margin was complicated by recognition that the dimensions that are hypothesised to show benefit, that is, acceptability to women and partners, and a reduction in costs appeal to different audiences—women will be primarily interested in acceptability and largely indifferent to costs (in a free at point of care NHS), whereas the potential reduction in costs will likely be the primary focus for the healthcare provider. We were also conscious that due to the inflation of the sample size due to (a) clustering, (b) losses due to non-matching in the propensity analysis and (c) loss to follow-up, the sample size for a smaller non-inferiority margin would quickly become not feasible within a realistic budget and timeframe. Given that, regardless of a superiority or non-inferiority design, any specific sample size will estimate the treatment effect to a certain level of precision (eg, the width of a 95% CI), we are confident that our final comparison group of 1920 in each arm (with ~115 NNU admissions in each arm (see sample size calculation below)), we will generate sufficient high-quality evidence to definitively answer the questions around safety, effectiveness, acceptability and cost-effectiveness for this important question

For the principal analysis of the primary outcome, we will use mixed effects logistic regression for the non-inferiority comparison of NNU admission within 48 hours of birth for 48 hours or longer (Yes/No). As sensitivity analyses to demonstrate that the estimated treatment effects are robust to the chosen method, we will also explore propensity score weighting (by inverse probability of receiving specified treatment) and single-stage regression, without using any propensity scoring, adjusting directly for the baseline factors relevant for treatment indication. We will also use propensity score matched (PSM) adjustment to control for treatment indication bias. The logistic model underlying the PSM will include variables such as age, Bishop’s score, previous vaginal birth, c-morbidities and relevant hospital-level factors, with 1:1 matching. Potential confounding variables will be identified before the start of the analysis, and these will be finalised after exploration of the data at the pilot stage, through the creation of directed acyclical graphs.

Similar analyses will be used for analyses of secondary outcomes, using logistic, linear, negative binomial and time-to-event regressions. For example, we will analyse the duration of hospital stay during IOL, time spent at home, total hospital stay and time to birth using linear models; while birth outwith hospital and breastfeeding will be analysed using logistic regression; and mode of birth using multinomial logistic regression.

For the remaining maternal secondary outcomes, we will include hyperstimulation, ≥1 induction agent, oxytocin use for induction or augmentation, maternal ICU/High dependency unit admission, haemorrhage, uterine rupture, pulmonary embolus and cardiorespiratory arrest. For the neonatal secondary outcomes, we will include meconium aspiration syndrome, respiratory support, neonatal infection, umbilical cord prolapse, neonatal birth trauma, neonatal encephalopathy (Grade II/III), therapeutic hypothermia and neonatal death. Logistic regression and Poisson or negative binomial regression, possibly inflated for excess zeros will be used as appropriate. For outcomes with a small number of events, we will use the appropriate exact regression procedure. As per the primary outcome, we will assess the influence of missing data for secondary outcomes using appropriate sensitivity-type analyses. We recognise that there are many secondary outcomes being analysed, as per the recommended core outcome set.^17^ We do not propose to make any formal statistical adjustment for the multiple comparisons. However, a caveat will be clearly expressed regarding the possibility of type 1 statistical error, given the multiple comparisons made. We will consider the following subgroup analyses, based on sufficient numbers to allow meaningful analyses: (a) nulliparous and parous women; (b) indication for IOL (post-dates IOL; maternal or clinician preference; maternal age and discomfort or social indication).

We propose the following sensitivity analyses: (a) within-site comparison of home versus in-hospital cervical ripening (restricted to sites that offer home cervical ripening), (b) per protocol analysis (women who actually are discharged home after commencing cervical ripening) and (c) complete case analysis to assess the effect of any strategies to deal with missing data.

Data from the larger cohort of women having IOL at 37+^0^ weeks’ gestation or more will be used to contextualise our findings on the background of unit practices and populations undergoing IOL. There is considerable inter-unit variation in both the rates of IOL and the risk profile of women giving birth, that needs to be considered. It will also allow us to capture any changes in practice over the study period regarding criteria for eligibility for home cervical ripening and change in method of IOL. This will help ensure the generalisability of our findings.

Future long-term outcome evaluation will be possible through data linkage to Hospital Episode Statistics and Scottish Morbidity Records.

### Missing data

We anticipate missing data, but estimate that no more than 10% of women will have missing usable data on primary outcome, eligibility, setting of cervical ripening and/or have some part of the baseline data (age, comorbidities and any relevant identified hospital-level factors). We will use evidence-based strategies to minimise any such losses and recover any missing data that is possible. We will monitor levels of missing data as the study progresses, identifying any outcomes or exposures and/or sites that are prone to missingness, and take corrective action (eg, additional feedback and support). We will conduct appropriate sensitivity type analyses, for example, using a multiple imputation approach assuming data are missing at random, and if the data warrant (eg, if there is differential missingness between the in-hospital and at-home cohorts) non-ignorable (informative) missing data generating mechanisms.

We will also conduct an exploratory analysis comparing the two methods of home IOL, that is, dinoprostone versus mechanical methods. We will use the same methods as outlined above for the primary and secondary outcomes in the overall analysis.

#### Sample size

The sample size is based on our principal analysis (women with singleton pregnancies having IOL at 39 weeks’ gestation or more) and primary comparison (home cervical ripening vs in-hospital cervical ripening with dinoprostone), estimated 6% NNU admission rate^1^ for babies born to mothers having IOL at >39 weeks’ gestation with no more than 4% excess NNU admission rate (from 6%), at 90% power, 2.5% one-sided alpha and an estimated ICC of 0.01. We will require 160 women in each of 12 sites (clusters) with uncomplicated pregnancies at 39 weeks or more undergoing IOL (total 1920 in each arm). To account for the fact that (a) only around 50% of women eligible for home cervical ripening in the intervention arm will actually initiate home cervical ripening, and (b) a larger pool of women is required in the control arm to allow for propensity score matching, our required sample size is 1920×2 (number of arms)/0.5 (numbers of women actually starting home cervical ripening and matching)/0.9 (for missing data), giving an overall required sample size of 8533.

Based on an estimate that 22% of all maternities have IOL at 39 weeks or more^1^ and that ~29% of these would be eligible for participation in our principal analysis (from scoping data from potential participating sites), and, in home cervical ripening sites ~50% of these will take up home cervical ripening, we anticipate achieving our recruitment targets within 20 months.

Current data from the National Maternity and Perinatal Audit (NMPA) for 2019 suggests that the national average rate of IOL after 37 weeks is 29.6%.^1^ As our proposed participating units have about 90 000 births per annum, we anticipate collecting data on approximately 41 000 women having IOL at 37 weeks’ gestation or more.

#### Participant identification and opt out

Participants will be identified from data recorded in specified fields in electronic maternity records. We will use data fields indicating IOL, estimated due date and date of IOL to identify women having IOL at 37 weeks’ gestation or more.

Women will be made aware of the CHOICE study through posters in participating sites; business cards; information leaflets; online adverts on hospital/maternity websites and relevant social media sites; and information in maternal electronic maternity records.

Women will be able to opt out of data provision by notifying their clinician or midwife, or emailing the study research midwife at the local site, and it will be recorded on their electronic record. There will be no restriction on co-enrolment in other studies.

#### Pilot phase

We propose a pilot phase to determine the parameters of the primary outcome and feasibility of obtaining the required sample size for analysis. This is based on the evaluable comparison group of 1920 women in each arm, so acts as an inherent check on home cervical ripening eligibility and uptake rates, the assumed level of missingness and attrition due to non-matching. We will assess variation of the primary outcome at the pilot stage, along with that of other measures of neonatal morbidity included as secondary outcomes (eg, any NNU admission and neonatal intensive care unit admission). We may redefine the parameters of NNU admission used in the primary outcome after analysis of pilot data, choosing the one with the lowest ICC or the one representing the least severe outcome, which has an ICC of 0.01 or less. This decision will be made in consultation between the expert project management group, the trial steering committee and the funder.

### CHOICE health economic analyses

Health economic analysis will be specified in a health economic analysis plan and reported in line with Consolidated Health Economic Evaluation Reporting Standards guidelines.^18^

Economic analyses will explore the cost effectiveness of at home versus inpatient cervical ripening for women undergoing IOL. Two separate cost-effectiveness questions will be addressed: (a) home cervical ripening with dinoprostone compared with in-hospital cervical ripening with dinoprostone and (b) home cervical ripening with balloon catheter compared with home cervical ripening with dinoprostone. The evaluation will involve within study cost-effectiveness analysis and a lifetime cost–utility analysis to account for any long-term impacts (cost and morbidity) of the alternative cervical ripening strategies. Resource use data will be obtained from the prospective multicentre observational cohort study using data obtained from the maternity information system, Badgernet Maternity and National Neonatal Research Database (NNRD) data.

Costs incurred by women and their families relating to IOL are relevant from the patient perspective and potentially important for the ‘at-home’ cervical ripening strategy. These data are not available from the observational datasets, and, therefore, tailored economic-related questions have been incorporated into a process evaluation survey described in section five below.

To account for bias in the observational data, methods such as multivariate regression and propensity scoring will be employed as recommended in guidelines for cost-effectiveness analysis based on observational data,^19 20^ which is consistent with the main study statistical analyses for this study. To capture any cost and morbidity events incurred in the neonatal period, the within-study analysis will include the primary study endpoint (NNU admission within 48 hours of birth for 48 hours or more) up to 1-month after birth. Outcomes will be reported as the incremental cost per NNU admission avoided (in line with primary study outcome) as well as incremental cost per birth up to 28 days after birth.

The lifetime analysis will account for longer term costs, quality of life, morbidity and disability from both the NHS and PSS and patient perspective and will report outcomes in terms of incremental cost per quality adjusted life years gained.

### Qualitative (q)CHOICE process evaluation

**Figure 1:**
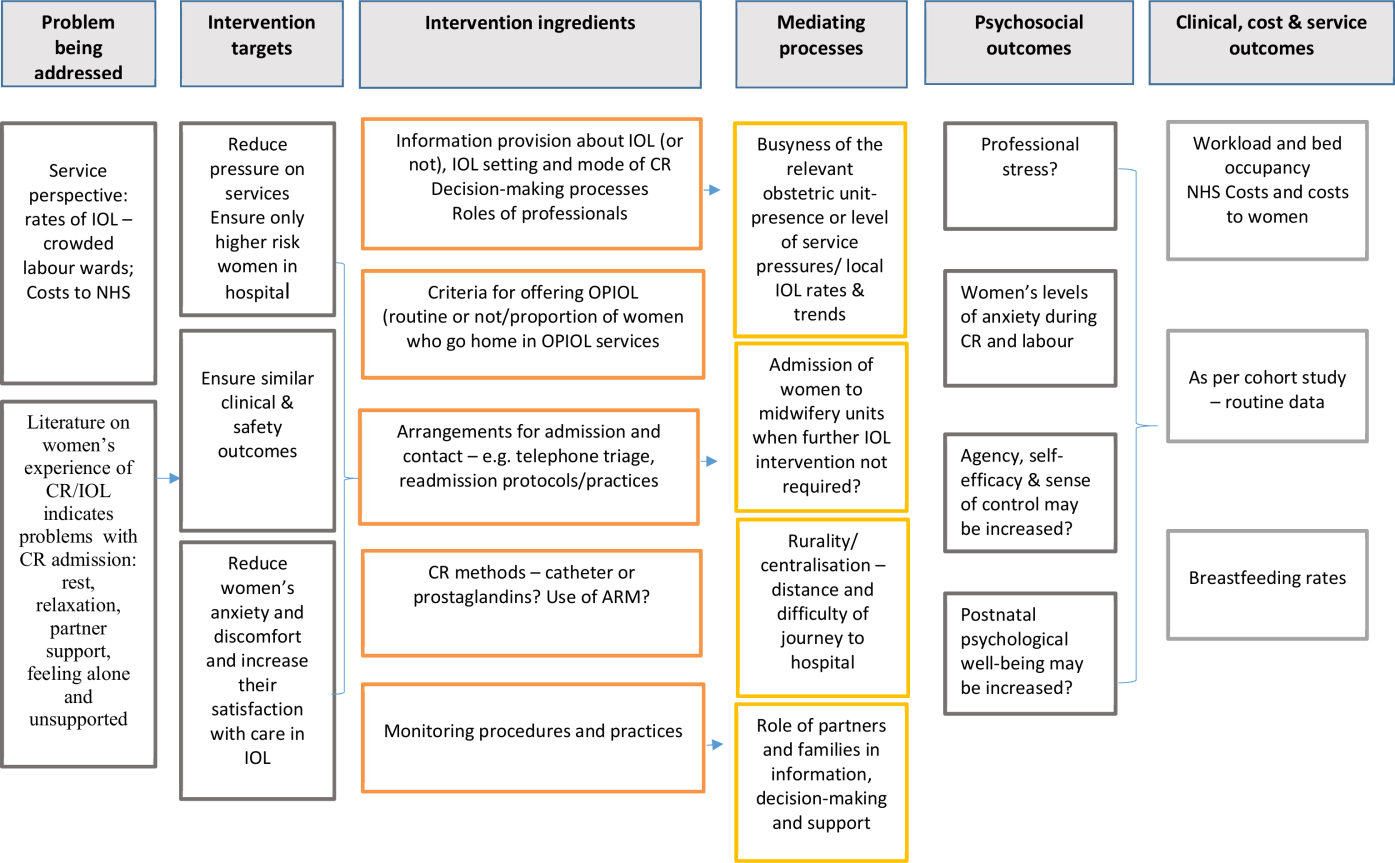
Logic model for the qCHOICE process evaluation. ARM, artificial rupture of membranes; CR, cervical ripening; IOL, induction of labour; NHS, National Health Service; OPIOL, Home cervical ripening; qCHOICE, qualitative cervical ripening at home or in-hospital—prospective cohort study and process evaluation.

The process evaluation, nested within the observational cohort study, will comprise of a questionnaire-based survey in at least 12 sites and 5 case studies. Both qualitative and quantitative data will be collected, specifically a women’s experience questionnaire, semi-structured interviews with women and birth partners, audio recordings of clinician/women consultations, interviews and focus-group discussions with professionals. Figure 1 describes the initial process evaluation logic model hypothesising the chain linking interventions and outcomes. This will inform data collection and analysis. At the final stage of data analysis, we will share and discuss emerging findings with a group of service users to develop a revised logic model and explanatory framework.

#### Questionnaire-based survey

Questionnaire data collection will take place over a 4–6-month period early in the study. CHOICE participating sites who use electronic maternity records accessible by women (the electronic equivalent of maternity hand-held records) will be invited to contribute to this part of the study. Women who have IOL at 39 weeks or more will receive a ‘push or SMS notification’ directing them to online study information when IOL is booked and a second notification around 10 days after they give birth. This will provide a link to the participant information sheets, consent form and online survey.

Push notifications are used by maternity services to prompt women to read information relating to their maternity care, including information within their electronic record. Women are able to opt out of SMS and push notifications, but routine monitoring of women’s use of their online record shows that a sufficient number of women use notifications and continue to access their record postnatally, thus enabling a broad sample to be reached.

The survey landing page will include a summary of qCHOICE and links to the information sheets before directing women to the consent questions, which they are asked to complete before completing the survey. A telephone number will be supplied for women to call if they have any questions about the survey, or to request a postal survey if preferred. Surveys will be submitted online via online surveys,^21^ by post or completed by phone with a member of the study team, with the support of an interpreter if needed. Participant contact details provided by survey respondents who are happy to be contacted further about a possible interview will be on a detachable back sheet of the questionnaire or a separate online page. Respondents will be informed that a £10 voucher will be offered to interview participants and that (with their consent) their birth partners may take part in an interview.

The survey will comprise validated tools as well as questions relating to service user costs, and the process of IOL as follows:

1. The Labour Agentry Scale (LAS; short form)^22^: the LAS is a well-established, validated measure of women’s experience during labour and birth. The short-form LAS includes 10 items with a 7-point Likert-type response. It measures perceived control during labour, which is the woman’s sense of mastery over internal and environmental factors and is highly correlated with satisfaction with care. The LAS will be the primary outcome.
2. A modified version of the IOL satisfaction questionnaire^23^ tested in the Balloon Catheter Versus Propess for Labour Induction (PROBIT-F) trial.^24^ This questionnaire focuses specifically on women’s experiences of aspects of IOL, including information, anxiety and physical and emotional discomfort.
3. The short-form Warwick-Edinburgh Mental Well-Being Scale (WEMWBS)^25^: a seven-item scale that measures mental well-being (as opposed to mental illness or disorder) representing positive attributes of well-being, including feeling and functioning.
4. Additional questions, which will inform the economic analysis from the woman’s perspective, will cover resource use and expenditures of cervical ripening for women, including number of returns and phone calls to hospital, time distance, mode of travel to and from hospital, partner role, additional expenditure on maternity items and medication while at home, and additional childcare expenditure (if any) while at home.
5. The survey will include demographic questions, questions about the process of IOL, questions relating to the impact of COVID-19 19 on their experience of IOL, a question asking women if they would be willing to be contacted regarding possible participation in a semi-structured interview and for permission for data linkage to the observational cohort study.

Survey data will be analysed using descriptive and inferential statistics. Descriptive statistics with 95% CIs will be reported for the total sample (by planned mode of cervical ripening—home or hospital, by actual mode (as some women who plan one mode may in practice have a different mode) and by study site). We will examine whether there are statistically significant differences in the primary outcome of sense of control (labour agentry) and by psychosocial outcome of postnatal psychological well-being score (WEMWBS) between women with home cervical ripening and women with in-hospital ripening. The covariates will include the reason for IOL, gestational age, maternal age, parity, sociodemographic status and ethnic group.

The sample size required to compare the experiences of women who had home and hospital cervical ripening is estimated to be 46 subjects within each of 12 sites (assuming equal numbers within each site), that is, 552 women in total. This is based on use of LAS^26^ where a change of 5.5 points is considered clinically meaningful. In an individually randomised study, to have 90% power at a 5% level of significance to detect an effect size of 0.5 (two-sided), we would need 85 evaluable subjects per arm (170 total). However, this has to be inflated for the clustering within each site. We assume that the intraclass correlation coefficient is 0.05 in this setting. We will also inflate the target sample by 10% to account for incomplete data/unusable questionnaires, aiming for 613 women in total.

We will invite all sites using accessible electronic maternity records to participate in the survey, but with the option to opt out. We will include at least 12 sites, that is, a total of at least 43 200 births annually across the sites. Our previous experience of questionnaire-based surveys, and the UK’s national maternity experience suggest a response rate of 40%. With an estimated eligibility of 22% of all maternities having IOL at 39 weeks or more, and 15% of these having home cervical ripening. We expect to achieve our sample size within 4 months and will monitor recruitment rates from each site and, if necessary, extend the survey period to ensure an adequate sample.

#### Case studies

In-depth case studies will be undertaken at five sites. The sample of five case studies is pragmatic, and selection is designed to balance depth with breadth of information and analysis with sites chosen to provide diversity and balance of service types on the basis of geography, service configuration and approaches to provision of IOL. We will undertake semi-structured interviews with women, their partners and a range of staff and stakeholders in each site. A topic guide and pathway mapping will be used to help focus the discussion. Interviews will explore perceptions and experiences of the service approach to induction and implementation of local cervical ripening protocols in practice.

##### Women and partner interviews

Women will be eligible for interview if they have IOL at a gestation of 39+0 weeks gestation or more, have given birth in one of the case study sites and responded to the survey indicating a willingness to be contacted regarding the interview.

A purposive sample of women will be included. A sampling frame will be constructed within and across case study sites with the aim of including a balance of nulliparous and parous women, women who were offered outpatient cervical ripening but declined, women who experienced this and women who were not offered it. The women approached will be given the opportunity to ask further questions and at least 1 week to decide whether to participate in an interview. Interviews will be conducted online or by telephone using a verbal consent protocol prior to the start of the interview. All women who consent to participate in an interview will also be asked whether they give consent for their birth partner to be invited for an interview. We anticipate interviewing between 10 and 15 women in each site (total 50–75 participants) and, assuming that around half of participants may have a birth partner willing to participate, we anticipate including around 25–38 birth partners. If couples express a preference to be interviewed together, this will be accommodated. ‘Birth partner’ will be defined by the women themselves.

##### Key professionals, stakeholders and maternity professionals’ interviews and focus groups

Key professionals and stakeholders for interviews will be identified with the support of the Principal Investigator for each local case study service but will typically include: head of midwifery, clinical director, consultant obstetricians and midwives, chairs of local Maternity Voices Partnerships, representatives from local maternity service user groups and service commissioners or health board leads. Interviews will be conducted online or by telephone. Verbal consent will be obtained at the start of the interview. We anticipate undertaking around 10 individual interviews in each case study site.

Midwives and obstetricians will be invited to participate in focus group discussions and we estimate that three focus groups comprising of 6–8 participants (total 18–24 participants) will be held in each site. These will be organised to facilitate participation of a diversity of maternity professionals, by including in a local audit meeting or study day. Focus groups may be held online or in person if access to the case study site is possible.

##### Observations of maternity visits discussing IOL

A small convenience sample of maternity visits will be included in each case study site in order to enable analysis of information provision and women’s information needs. Up to five maternity professionals in each site will be provided with a digital recorder and given instructions on use and asked to record three consecutive interviews with the woman’s consent. We will follow-up the recorded consultations with a brief (up to 10 min) telephone interview to explore the woman’s understanding of the information provided.

##### Qualitative data analysis

All qualitative data will be transcribed and entered into the analysis support software NVivo to support data management and analysis. Documentary sources will be added to the NVivo project file as PDF files. Visual approaches will be used to support the discussion and analysis of the pathways. Recordings of discussions will be analysed using a structured approach to conversation analysis. Interviews with women, partners and health professionals will be transcribed and analysed using a thematic framework approach, based on frameworks developed in the recent work by the study team as part of the PROBIT-F trial.^8 27^

## Research ethics approval and dissemination

CHOICE has National Research Ethics Service Committee approval (York and Humber—Sheffield Research Ethics Committee, REC reference: 20/YH/0145), National R&D approval in Scotland (NHS Research Scotland Permissions) and England (Health Research Authority), and approval from the Public Benefit and Privacy Panel in Scotland is pending. The study will be conducted in accordance with the principles of the International Conference on Harmonisation Tripartite Guideline for Good Clinical Practice.

Results will be submitted for peer-reviewed academic publication and presented at international conferences. Meta-data produced in this study will also become available to Health Data Research UK Gateway. STROBE guidance^16^ and RECORD guidance^15^ will be used to guide transparent reporting.

## Supplementary Material

Reviewer comments

Author's manuscript
