# Supplementary material for: Cervical ripening at home or in-hospital—prospective cohort study and process evaluation (CHOICE) study: a protocol
Source: BMJ Open. 2021 May 4;11(5):e050452. doi: 10.1136/bmjopen-2021-050452 (PMC8098973; doi:10.1136/bmjopen-2021-050452)

BMJ Open is committed to open peer review. As part of this commitment we make the peer review history of every article we publish publicly available.

When an article is published we post the peer reviewers' comments and the authors' responses online. We also post the versions of the paper that were used during peer review. These are the versions that the peer review comments apply to.

The versions of the paper that follow are the versions that were submitted during the peer review process. They are not the versions of record or the final published versions. They should not be cited or distributed as the published version of this manuscript.

BMJ Open is an open access journal and the full, final, typeset and author-corrected version of record of the manuscript is available on our site with no access controls, subscription charges or pay-per-view fees (http://bmjopen.bmj.com).

If you have any questions on BMJ Open's open peer review process please email info.bmjopen@bmj.com

BMJ Open

Cervical ripening at home or in-hospital - prospective cohort study and process evaluation (CHOICE) study: Protocol

Journal:	*BMJ Open*
Manuscript ID	bmjopen-2021-050452
Article Type:	Protocol
Date Submitted by the Author:	19-Feb-2021
Complete List of Authors:	Stock, Sarah; The University of Edinburgh<br>Bhide, Amarnath; St George's University of London<br>Richardson, Heather; NHS Lothian University Hospitals Division<br>Black, Mairead; University of Aberdeen<br>Yuill, Cassandra; City University of London<br>Harkness, Mairi; University of Stirling<br>Reid, Maggie; The University of Edinburgh<br>Wee, Fiona; The University of Edinburgh<br>Cheyne, Helen; University of Stirling<br>McCourt, Christine; City University, City University London<br>Rana, Dikshyanta; University of Glasgow, Institute of Health and Wellbeing<br>Boyd, Kathleen; University of Glasgow, Health Economics & Health Technology Assessment<br>Sanders, Julia; Cardiff University, School of Healthcare Sciences<br>Heera, Neelam; Patient and Public Involvement Representative<br>Huddleston, Jane; Patient and Public Involvement Representative<br>Denison, Fiona; The University of Edinburgh, MRC Centre for Reproductive Healht<br>Pasupathy, Dharmintra; The University of Sydney<br>Modi, Neena; Imperial College London, Medicine<br>Smith, Gordon; University of Cambridge, Obstetrics and Gynaecology<br>Norrie, John ; The University of Edinburgh
Keywords:	Maternal medicine < OBSTETRICS, Fetal medicine < OBSTETRICS, OBSTETRICS

SCHOLARONE™
Manuscripts

I, the Submitting Author has the right to grant and does grant on behalf of all authors of the Work (as defined in the below author licence), an exclusive licence and/or a non-exclusive licence for contributions from authors who are: i) UK Crown employees; ii) where BMJ has agreed a CC-BY licence shall apply, and/or iii) in accordance with the terms applicable for US Federal Government officers or employees acting as part of their official duties; on a worldwide, perpetual, irrevocable, royalty-free basis to BMJ Publishing Group Ltd ("BMJ") its licensees and where the relevant Journal is co-owned by BMJ to the co-owners of the Journal, to publish the Work in this journal and any other BMJ products and to exploit all rights, as set out in our licence.

The Submitting Author accepts and understands that any supply made under these terms is made by BMJ to the Submitting Author unless you are acting as an employee on behalf of your employer or a postgraduate student of an affiliated institution which is paying any applicable article publishing charge ("APC") for Open Access articles. Where the Submitting Author wishes to make the Work available on an Open Access basis (and intends to pay the relevant APC), the terms of reuse of such Open Access shall be governed by a Creative Commons licence – details of these licences and which Creative Commons licence will apply to this Work are set out in our licence referred to above.

Other than as permitted in any relevant BMJ Author's Self Archiving Policies, I confirm this Work has not been accepted for publication elsewhere, is not being considered for publication elsewhere and does not duplicate material already published. I confirm all authors consent to publication of this Work and authorise the granting of this licence.

For peer review only

Cervical ripening at home or in-hospital - prospective cohort study and process evaluation (CHOICE) study: Protocol

Sarah J Stock, Usher Institute, University of Edinburgh NINE Edinburgh BioQuarter, 9 Little France Road, Edinburgh EH16 4UX UK sarah.stock@ed.ac.uk +44 (0)7894629934 (corresponding author) ORCID ID 0000-0003-4308-856X

Dr Amarnath Bhide, Consultant Obstetrician, Obstetrics and Gynaecology, St George's University Hospitals Trust, London, UK. ORCID ID: 0000-3-2393-7501

Heather Richardson, Subspecialty Registrar in Maternal and Fetal Medicine, Simpson Centre for Reproductive Health, NHS Lothian, Edinburgh, UK

Dr Mairead Black, Senior Clinical Lecturer, Institute of Applied Health Sciences, University of Aberdeen, Aberdeen, UK

Dr Cassandra Yuill, Research Fellow, Centre for Maternal & Child Health Research School of Health Sciences, City, University of London, London, UK

Dr Mairi Harkness, Nursing, Midwifery and Allied Health Professions Research Unit (NMAHP), Research Fellow, University of Stirling, Stirling, UK

Mrs Maggie Reid, Research Midwife, Usher Institute, University of Edinburgh, Edinburgh, UK

For peer review only

Mrs Fiona Wee, Trial Manager, Usher Institute, University of Edinburgh, Edinburgh, UK

Professor Helen Cheyne, Royal College of Midwives Professor of Midwifery, Nursing Midwifery and Allied Health Professions Research Unit (NMAHP), University of Stirling, Stirling, UK. ORCID ID: 0000-0001-5738-8390

Professor Christine McCourt, Professor of Maternal and Child Health, School of Health Sciences, City, University of London, London, UK

Miss Dikshyanta Rana, Research Assistant, Institute of Health and Well Being, University of Glasgow, Glasgow, UK

Dr Kathleen Boyd, Senior Lecturer, Institute of Health and Well Being, University of Glasgow, Glasgow, UK

Professor Julia Sanders, Professor of Clinical Nursing & Midwifery, School of Healthcare Sciences, Cardiff University, Cardiff, UK

Miss Neelam Heera, Patient and Public Involvement Representative

Mrs Jane Huddleston, Patient and Public Involvement Representative

Professor Fiona Denison, Reader/Honorary Consultant in Maternal and Fetal Medicine, MRC Centre for Reproductive Health, University of Edinburgh, Edinburgh, UK

For peer review only

Professor Dharmintra Pasupathy, Professor of Maternal Fetal Medicine, Faculty of Medicine and Health, University of Sydney, Sydney, Australia

Professor Neena Modi, Professor of Neonatal Medicine, School of Public Health, Imperial College London, London, UK

Professor Gordon C S Smith, Professor of Obstetrics and Gynaecology, Department of Obstetrics and Gynaecology, The University of Cambridge, Cambridge, UK

Professor John Norrie, Professor of Medical Statistics and Trial Methodology / Director of Edinburgh Clinical Trials Unit / Co-Head of Centre for Population Health Sciences, Usher Institute, University of Edinburgh, Edinburgh, UK

Word count: 6222

Key words: Induction of labour, Prostaglandin, Cervical Ripening Balloon, Pregnancy

For peer review only

ABSTRACT

Introduction

The aim of the CHOICE study is to compare home versus in-hospital cervical ripening to determine; whether home cervical ripening is safe (for the primary outcome of neonatal unit [NNU] admission), acceptable to women, and cost-effective from the perspective of both women and the NHS.

Methods and analysis

We will perform a prospective multicentre observational cohort study with an internal pilot phase. We will obtain data from electronic health records from at least 14 maternity units offering only in-hospital cervical ripening and 12 offering dinoprostone home cervical ripening. We will also conduct a cost-effectiveness analysis and a mixed methods study to evaluate processes and women/partner experiences. Our primary sample size is 8,533 women with singleton pregnancies undergoing induction of labour (IOL) at 39^{+0} weeks' gestation or more. To achieve this and contextualise our findings, we will collect data relating to a cohort of approximately 41,000 women undergoing IOL after 37 weeks.

We will use mixed effects logistic regression for the non-inferiority comparison of neonatal unit admission and propensity score matched adjustment to control for treatment indication bias.

The economic analysis will be undertaken from the perspective of the National Health and Personal Social Services (NHS and PSS) and the pregnant woman. It will include a within-study cost-effectiveness analysis and a lifetime cost-utility analysis to account for any long-term impacts of the cervical ripening strategies. Outcomes will be reported as incremental cost

For peer review only

per NNU admission avoided and incremental cost per quality adjusted life year (QALY) gained.

Research Ethics approval and Dissemination

CHOICE has been funded and approved by the National Institute of Healthcare Research Health Technology and Assessment (NIHR HTA), and the results will be disseminated via publication in peer reviewed journals.

ARTICLE SUMMARY

Strengths and limitations of this study

- This is a large study to evaluate the safety of at-home cervical ripening
- We will set up a platform for data collection using electronic health records to enable future research into rare safety outcomes
- The study includes assessment of women's views and experiences using validated questionnaires as well as qualitative methods.
- Observational design of the study makes it vulnerable to residual confounding.

For peer review only

INTRODUCTION

Induction of labour (IOL) is the most common obstetric intervention offered to women when risks of continuing the pregnancy are thought to outweigh risks of birth. Increases in IOL rates over the past 10 years mean that now 29.6% of all pregnant women in the UK have their labour induced.(1) IOL at term, when compared to expectant management of pregnancy, reduces caesarean birth, maternal hypertensive disease and complications,(2) as well as being associated with a reduction in perinatal mortality.(3, 4) The demands on maternity services are increasing to accommodate increasing rates of IOL.(5) Although IOL (compared to expectant management) reduces overall hospital stay, it increases the time on labour and delivery wards,(2) having a major impact on resources, staffing, and negatively impacts on women's experience of labour.(6-8)

Cervical ripening is a key component of IOL.(9) It may initiate labour, but is often followed by artificial rupture of membranes +/- intravenous oxytocin infusion. NICE guidance(10) recommends pre-induction cervical ripening in all women having IOL unless there is a contraindication.

Traditionally cervical ripening has been performed entirely in-hospital, to allow monitoring of maternal/fetal wellbeing and early recognition of complications.(11) However, an increasing number of UK maternity units offer outpatient (home) cervical ripening. As the rate of IOL is increasing, home cervical ripening may provide opportunities to reduce the burden on the NHS, for example, by reducing hospital stay during IOL. However, the safety and acceptability of home cervical ripening have not been fully evaluated. NICE(10) identified the need to assess the safety, efficacy and clinical and cost-effectiveness of outpatient and inpatient IOL in the UK setting, taking into account women's views. A recent Cochrane review found insufficient evidence to draw conclusions on the efficacy, safety and cost-effectiveness of home-induction

For peer review only

of labour and indicated that large prospective cohort studies would be needed.(12) Maternity service users have identified IOL as an important research topic,(13) and women have reported specific negative experiences such as increased pain and anxiety and lack of support which may be alleviated by home cervical ripening.(8) Potential NHS cost savings of home cervical ripening could be offset by increased costs of any additional morbidity resulting from home cervical ripening, costs to parents may be increased, and acceptability of home cervical ripening is unknown. Health services need to balance the full resource impact of IOL with the need to provide safe and acceptable care.

In the cervical ripening at home or in-hospital – prospective cohort study and process evaluation (CHOICE) study, we will perform an observational cohort study with a cost-effectiveness analysis and process evaluation to address the question "Is it safe, effective, cost-effective and acceptable to women to carry out home cervical ripening during IOL?" These analyses will provide information to help women and their caregivers make informed decisions around how to have IOL.

Our main aim is to compare the setting of cervical ripening at home versus in-hospital. As the NICE recommended agent for cervical ripening is vaginal prostaglandin, our primary comparison will be home dinoprostone versus in-hospital dinoprostone. In order to future-proof the study, we will include a secondary comparison: home cervical ripening with balloon catheter versus home cervical ripening with dinoprostone. By including two different methods of home cervical ripening within our study, we will provide initial comparative evidence on these two methods of home labour induction.

For peer review only

METHODS AND ANALYSIS

CHOICE Prospective Cohort study

Study design and setting

We will carry out a prospective multicentre cohort study using de-identified clinical data from electronic hospital records. The primary outcome will be non-inferiority of NNU/special care admission for 48 hours or longer, initiated within 48 hours of birth.

The study will be performed in at least 26 UK obstetric units, 14 of which offer exclusively in-hospital cervical ripening and 12 offer dinoprostone cervical ripening both in-hospital and at home.

Participating maternity units will be purposively selected to represent the diverse range of maternity service settings in the UK, and include urban tertiary referral units, mid-sized urban district general hospitals and small, more isolated, rural units.

Data sources

Data will be collected directly from electronic maternity (and neonatal) records for participants who had babies admitted to a neonatal unit. These data are recorded by clinical staff (midwives, doctors and neonatal nurses) during the course of antenatal, intrapartum and postpartum care. Existing data fields, supplemented by new bespoke, data entry fields enabled in the maternity dataset at participating sites will be used. Unless women opt-out of secondary data use (from similar studies we estimate <1% will opt out), de-identified data will be transferred from participating sites to a secure University of Edinburgh server for analysis.

For peer review only

No personal data will be collected. Potentially identifiable data, such as the date and time of birth, date of events such as commencing cervical ripening, hospital discharge, will be converted into gestation at birth (weeks + days); and antenatal and postnatal events into "t–x" and "t+x" hours and days respectively.

Population, inclusion and exclusion criteria

We will initially apply broad inclusion criteria and collect data from all women having IOL at 37^{+0} weeks gestation or more to create a cohort for analyses. Women who have opted out of data provision will be excluded.

We will then apply more stringent inclusion and exclusion criteria at the analysis stage for a suite of nested analyses. In our primary analysis, we will create a cohort of women with "uncomplicated" (ie those with no identified risk factors for adverse maternal or perinatal outcomes defined below) pregnancies in whom there was no contraindication to home cervical ripening, who had singleton pregnancies with IOL at 39 weeks gestation or more. This group will include women having IOL for post-dates, but also women having IOL because of maternal or clinician preference, IOL for maternal age, IOL for discomfort or social indications. Exclusion criteria will consist of grand multiparity (6 or more previous births), previous caesarean section, antepartum stillbirth (before cervical ripening initiated), Class III obesity at booking (BMI 40 kg/m^2 or more), Prelabour rupture of membranes (ROM) documented as primary or other indication for IOL (prolonged ROM; Spontaneous ROM; Suspected Spontaneous ROM), Maternal or fetal condition that would or could preclude home cervical ripening documented as primary or other indication for IOL (Maternal conditions: proteinuria; hypertension; antepartum haemorrhage; diabetes; obstetric cholestasis; past obstetric history; pre-eclampsia; PIH/PET (not defined); PIH; PET; thrombophilia. Fetal conditions: oligohydramnios; reduced liquor volume; macrosomia;

For peer review only

intrauterine growth restriction (IUGR); static growth; congenital fetal anomaly; polyhydramnios; abnormal CTG/Doppler; breech; reduced fetal movements; termination of pregnancy for fetal anomaly).

We will explore the potential for additional analyses which may include IOL for other indications (e.g. reduced fetal movements) or in other populations (e.g. multiple pregnancies; women with a previous caesarean birth). However, in general, home cervical ripening is only offered to 'low risk' women, so we anticipate that numbers of higher risk women having home IOL may not be high enough for meaningful analyses.

Exposure and Outcomes

Our primary aim is to compare home versus in-hospital cervical ripening. We will collect data at individual-level. The exposure group will be women who, at the start of the cervical ripening process, plan to have home cervical ripening. The comparator group will be women who planned to have in-hospital cervical ripening from maternity units not offering home cervical ripening. This will minimise potential bias arising from the fact that, in maternity units which offer both home and in-hospital cervical ripening, the risk of complications in the babies of women having home cervical ripening (lower risk pregnancies) is inherently different to that of babies of women having in-hospital cervical ripening (higher risk pregnancies).

As the NICE recommended agent for cervical ripening is vaginal prostaglandin, our primary comparison will be home dinoprostone versus in-hospital dinoprostone. Dinoprostone is now most commonly administered as 10mg slow-release pessary (Propess, Ferring) which stays in place for 24 hours. We will use this formulation in our primary comparison.

We will include a secondary exploratory comparison - home cervical ripening with balloon catheter (exposure) versus home cervical ripening with dinoprostone (comparator), to explore

For peer review only

if there are any indications of different safety profiles of these two methods of home cervical ripening.

Our proposed primary outcome will be admission to a NNU/Special care baby unit for 48 hours or longer, initiated within 48 hours of birth. NNU admission is a marker of neonatal morbidity and is the leading core outcome defined for studies of IOL.(14) Any increase in NNU admission of term babies is undesirable due to the separation of mother and baby. However, NNU admission rates are highly variable between maternity units and are likely to depend on local policies and culture. We therefore, plan to use a primary outcome which represents more severe neonatal morbidity (admission to a NNU within 48 h of birth for 48 h or longer) which is less likely to be influenced by site-specific factors. We may re-define the parameters of NNU admission used in the primary outcome after analysis of pilot data (see section on 'Pilot phase').

We have prespecified a number of secondary outcomes to assess the safety of home cervical ripening with respect to neonatal and maternal morbidity, shown in Table 1. These include outcomes from a core outcome set for studies of IOL.(14) We will also include secondary outcomes relating to the effectiveness of home cervical ripening, to explore whether the setting of cervical ripening influences subsequent labour and birth. Mother and baby outcomes were suggested by our lay consultation as important to include. We will use birthweight, birthweight centile, small for gestational age and large for gestational age as parameters to check the validity of our matching procedures in analyses. Birthweight is an objective outcome that may represent pregnancy complications, but extremely unlikely to be affected by the setting of cervical ripening. Comparison of birthweights between groups should provide reassurance that we have minimised systemic bias in our analyses.

For peer review only

Statistical analysis

All analyses will be fully specified in a comprehensive Statistical Analysis Plan and agreed by the Steering Committee. Analyses will be carried out in accordance with relevant guidance including RECORD(15) and STROBE.(16)

We will include at least 14 maternity units offering only in-hospital cervical ripening and 12 offering dinoprostone home cervical ripening (~95,000 deliveries per annum). We will invite additional maternity units to opt in to data provision, to allow contingency in case of 'cross overs' due to sites changing their IOL protocols during the study period.

We considered a superiority design for CHOICE, but decided against it because i) safety is a key concern to both clinicians and women, and was specified as the important outcome in the commissioning brief; ii) it is not plausible to hypothesise that home cervical ripening (intervention) is safer than in-hospital cervical ripening (comparator – the standard of care); and iii) it is not ethical to use a superiority design to test an intervention which may be worse (in terms of safety) than the established standard. Therefore, a non-inferiority design was chosen with a non-inferiority margin of 4% (deemed as likely to be an important difference on consultation with women and clinicians) for the primary outcome of neonatal unit admission.

Establishing the appropriate non-inferiority margin was complicated by recognition that the dimensions that are hypothesised to show benefit, i.e. acceptability to women and partners, and a reduction in costs appeal to different audiences – women will be primarily interested in acceptability and largely indifferent to costs (in a free at point of care NHS), whereas the potential reduction in costs will likely be the primary focus for the healthcare provider. We were also conscious that due to the inflation of the sample size due to (a) clustering; (b) losses due to non-matching in the propensity analysis and (c) loss to follow up, the sample size for a

For peer review only

smaller non-inferiority margin would quickly become not feasible within a realistic budget and timeframe. However, given that, regardless of a superiority or non-inferiority design, any specific sample size will estimate the treatment effect to a certain level of precision (e.g. the width of a 95% confidence interval), we are confident that our final comparison group of 1,920 in each arm (with ~ 115 NNU admissions in each arm [see sample size calculation below]), we will generate sufficient high-quality evidence to definitively answer the questions around safety, effectiveness, acceptability, and cost-effectiveness for this important question

For the principal analysis of the primary outcome, we will use mixed effects logistic regression for the non-inferiority comparison of NNU admission within 48 hours of birth for 48 hours or longer (Yes/No). As sensitivity analyses to demonstrate that the estimated treatment effects are robust to the chosen method, we will also explore propensity score weighting (PSW by inverse probability of receiving specified treatment) and single-stage regression, without using any propensity scoring, adjusting directly for the baseline factors relevant for treatment indication. We will also use propensity score matched (PSM) adjustment to control for treatment indication bias. The logistic model underlying the PSM will include variables such as age, Bishop's score, previous vaginal birth, co-morbidities, and relevant hospital-level factors, with 1:1 matching. Potential confounding variables will be identified before the start of the analysis, and these will be finalised after exploration of the data at the pilot stage, through the creation of directed acyclical graphs.

Similar analyses will be used for analyses of secondary outcomes, using logistic, linear, negative binomial, and time-to-event regressions. For example, we will analyse the duration of hospital stay during IOL, time spent at home, total hospital stay, and time to birth using linear models; while birth outwith hospital and breastfeeding will be analysed using logistic regression; and mode of birth using multinomial logistic regression.

For peer review only

For peer review only - http://bmjopen.bmj.com/site/about/guidelines.xhtml

For the remaining maternal secondary outcomes, we will include hyperstimulation, ≥1 induction agent, oxytocin use for induction or augmentation, maternal ICU/HDU admission, haemorrhage, uterine rupture, pulmonary embolus, and cardio-respiratory arrest. For the neonatal secondary outcomes, we will include meconium aspiration syndrome, respiratory support, neonatal infection, umbilical cord prolapse, neonatal birth trauma, neonatal encephalopathy (Grade II/III), therapeutic hypothermia and neonatal death. Logistic regression and Poisson or negative binomial regression, possibly inflated for excess zeros will be used as appropriate. For outcomes with a small number of events, we will use the appropriate exact regression procedure. As per the primary outcome, we will assess the influence of missing data for secondary outcomes using appropriate sensitivity-type analyses. We recognise that there are many secondary outcomes being analysed, as per the recommended core outcome set (17). We do not propose to make any formal statistical adjustment for the multiple comparisons. However, a caveat will be clearly expressed regarding the possibility of type 1 statistical error, given the multiple comparisons made. We will consider the following subgroup analyses, based on sufficient numbers to allow meaningful analyses: 1) nulliparous and parous women; 2) indication for IOL (post-dates IOL; maternal or clinician preference; maternal age; discomfort or social indication)

We propose the following sensitivity analyses. 1) Within-site comparison of home versus in-hospital cervical ripening (restricted to sites that offer home cervical ripening) 2) Per protocol analysis (women who actually are discharged home after commencing cervical ripening) 3) Complete case analysis to assess the effect of any strategies to deal with missing data.

Data from the larger cohort of women having IOL at 37^{+0} weeks' gestation or more, will be used to contextualise our findings on the background of unit practices and populations undergoing IOL. There is considerable inter-unit variation in both the rates of IOL and the risk

For peer review only

profile of women giving birth, that need to be considered. It will also allow us to capture any changes in practice over the study period regarding criteria for eligibility for home cervical ripening and change in method of IOL. This will help ensure the generalisability of our findings.

Future long-term outcome evaluation will be possible through data linkage to Hospital Episode Statistics and Scottish Morbidity Records.

Missing data

We anticipate missing data, but estimate that no more than 10% of women will have missing usable data on primary outcome, eligibility, setting of cervical ripening and/or have some part of the baseline data (age, co-morbidities, and any relevant identified hospital-level factors). We will use evidence-based strategies to minimise any such losses and recover any missing data that is possible. We will monitor levels of missing data as the study progresses, identifying any outcomes or exposures and/or sites that are prone to missingness, and take corrective action (e.g. additional feedback and support). We will conduct appropriate sensitivity type analyses, for example, using a multiple imputation approach assuming data are missing at random; and, if the data warrant (for example, if there is differential missingness between the in-hospital and at-home cohorts) non-ignorable (informative) missing data generating mechanisms.

We will also conduct an exploratory analysis comparing the two methods of home IOL, i.e. dinoprostone vs. mechanical methods. We will use the same methods as outlined above for the primary and secondary outcomes in the overall analysis.

For peer review only

Sample size

The sample size is based on our principal analysis (women with singleton pregnancies having IOL at 39 weeks gestation or more) and primary comparison (home cervical ripening vs in-hospital cervical ripening with dinoprostone), estimated 6% NNU admission rate (1) for babies born to mothers having IOL at >39 weeks gestation with no more than 4% excess NNU admission rate (from 6%), at 90% power, 2.5% 1-sided alpha, and an estimated ICC of 0.01. We will require 160 women in each of 12 sites (clusters) with uncomplicated pregnancies at 39 weeks or more undergoing IOL (total 1,920 in each arm). To account for the fact that i) only around 50% of women eligible for home cervical ripening in the intervention arm will actually initiate home cervical ripening, and ii) a larger pool of women is required in the control arm to allow for propensity score matching, our required sample size is 1,920 *2 (number of arms) / 0.5 (numbers of women actually starting home cervical ripening and matching) / 0.9 (for missing data), giving an overall required sample size of 8,533.

Based on an estimate that 22% of all maternities have IOL at 39 weeks or more(1) and that ~29% of these would be eligible for participation in our principal analysis (from scoping data from potential participating sites), and, in home cervical ripening sites ~50% of these will take up home cervical ripening, we anticipate achieving our recruitment targets within 20 months.

Current data from the NMPA for 2019 suggests that the national average rate of IOL after 37 weeks is 29.6%.(1) As our proposed participating units have about 90,000 births per annum, we anticipate collecting data on approximately 41,000 women having IOL at 37 weeks gestation or more.

For peer review only

Participant identification and opt out

Participants will be identified from data recorded in specified fields in electronic maternity records. We will use data fields indicating IOL, estimated due date (EDD) and date of IOL to identify women having IOL at 37 weeks gestation or more.

Women will be made aware of the CHOICE study through posters in participating sites; business cards; information leaflets; online adverts on hospital/maternity websites and relevant social media sites; and information in maternal electronic maternity records.

Women will be able to opt out of data provision by notifying their clinician or midwife, or emailing the study research midwife at the local site, and it will be recorded on their electronic record. There will be no restriction on co-enrolment in other studies.

Pilot phase

We propose a pilot phase to determine the parameters of the primary outcome and feasibility of obtaining the required sample size for analysis. This is based on the evaluable comparison group of 1,920 women in each arm, so acts as an inherent check on home cervical ripening eligibility and uptake rates, the assumed level of missingness and attrition due to non-matching. We will assess variation of the primary outcome at the pilot stage; along with that of other measures of neonatal morbidity included as secondary outcomes (e.g. any NNU admission, NICU admission). We may redefine the parameters of NNU admission used in the primary outcome after analysis of pilot data, choosing the one with the lowest ICC, or the one representing the least severe outcome which has an ICC of 0.01 or less. This decision will be made in consultation between the expert project management group, the trial steering committee (TSC) and the funder.

For peer review only

CHOICE Health Economic Analyses

Health economic analysis will be specified in a health economic analysis plan and reported in line with CHEERS guidelines.(18)

Economic analyses will explore the cost-effectiveness of at home versus inpatient cervical ripening for women undergoing IOL. Two separate cost-effectiveness questions will be addressed: (i) home cervical ripening with dinoprostone compared to in-hospital cervical ripening with dinoprostone and (ii) home cervical ripening with balloon catheter compared to home cervical ripening with dinoprostone. The evaluation will involve within study cost-effectiveness analysis and a lifetime cost-utility analysis to account for any long-term impacts (cost and morbidity) of the alternative cervical ripening strategies. Resource use data will be obtained from the prospective multicentre observational cohort study using data obtained from the maternity information system, Badgernet Maternity and National Neonatal Research Database (NNRD) data.

Costs incurred by women and their families relating to IOL are relevant from the patient perspective and potentially important for the 'at-home' cervical ripening strategy. These data are not available from the observational datasets, and therefore tailored economic-related questions have been incorporated into a process evaluation survey described in section 5 below.

To account for bias in the observational data, methods such as multivariate regression and propensity scoring will be employed as recommended in guidelines for cost-effectiveness analysis based on observational data,(19,20) which is consistent with the main study statistical analyses for this study. To capture any cost and morbidity events incurred in the neonatal period, the within-study analysis will include the primary study endpoint (NNU admission within 48 hours of birth for 48 hours or more) up to one-month post-birth. Outcomes will be

For peer review only

reported as the incremental cost per NNU admission avoided (in line with primary study outcome) as well as incremental cost per birth up to 28 days post-birth.

The lifetime analysis will account for longer term costs, quality of life, morbidity and disability from both the NHS & PSS and patient perspective and will report outcomes in terms of incremental cost per Quality Adjusted Life Years (QALYs) gained.

Qualitative (q)CHOICE Process evaluation

The process evaluation, nested within the observational cohort study, will comprise of a questionnaire-based survey in at least 12 sites and five case studies. Both qualitative and quantitative data will be collected, specifically a women's experience questionnaire, semi-structured interviews with women and birth partners, audio recordings of clinician/women consultations, interviews and focus-group discussions with professionals. Figure 1 describes the initial process evaluation logic model hypothesising the chain linking interventions and outcomes. This will inform data collection and analysis. At the final stage of data analysis, we will share and discuss emerging findings with a group of service users to develop a revised logic model and explanatory framework.

Questionnaire-based survey

Questionnaire data collection will take place over a four- to six-month period early in the study. CHOICE participating sites who use electronic maternity records accessible by women (the electronic equivalent of maternity hand-held records), will be invited to contribute to this part of the study. Women who have IOL at 39 weeks or more will receive a 'push or SMS notification' directing them to online study information when IOL is booked and a second notification around 10 days after they give birth. This will provide a link to the participant information sheets, consent form and online survey.

For peer review only

Push notifications are used by maternity services to prompt women to read information relating to their maternity care, including information within their electronic record. Women are able to opt out of SMS and push notifications, but routine monitoring of women's use of their online record shows that a sufficient number of women use notifications and continue to access their record postnatally, thus enabling a broad sample to be reached.

The survey landing page will include a summary of qCHOICE and links to the information sheets before directing women to the consent questions, which they are asked to complete before completing the survey. A telephone number will be supplied for women to call if they have any questions about the survey, or to request a postal survey if preferred. Surveys will be submitted online via Online Surveys,(21) by post or completed by phone with a member of the study team, with the support of an interpreter if needed. Participant contact details provided by survey respondents who are happy to be contacted further about a possible interview will be on a detachable back sheet of the questionnaire or a separate online page. Respondents will be informed that a £10 voucher will be offered to interview participants and that (with their consent) their birth partners may take part in an interview.

The survey will comprise validated tools as well as questions relating to service user costs, and the process of IOL as follows:

1. The Labour Agentry Scale (short form).(22) The LAS is a well-established, validated measure of women's experience during labour and birth. The short form LAS includes 10 items with a 7-point Likert type response. It measures perceived control during labour, which is the woman's sense of mastery over internal and environmental factors and is highly correlated with satisfaction with care. The LAS will be the primary outcome.

For peer review only

2. A modified version of the IOL satisfaction questionnaire(23) tested in the PROBIT-F trial. This questionnaire focuses specifically on women's experiences of aspects of IOL including information, anxiety and physical and emotional discomfort.

3. The short form Warwick-Edinburgh mental wellbeing scale WEMWBS.(24) A seven-item scale that measures mental wellbeing (as opposed to mental illness or disorder) representing positive attributes of wellbeing including feeling and functioning.

4. Additional questions which will inform the economic analysis from the woman's perspective will cover resource use and expenditures of cervical ripening for women including number of returns and phone calls to hospital, time distance, mode of travel to and from hospital, partner role, additional expenditure on maternity items and medication while at home, and additional childcare expenditure (if any) while at home.

5. The survey will include demographic questions, questions about the process of IOL, questions relating to the impact of COVID 19 on their experience of IOL, a question asking women if they would be willing to be contacted regarding possible participation in a semi-structured interview and for permission for data linkage to the observational cohort study.

Survey data will be analysed using descriptive and inferential statistics. Descriptive statistics with 95% confidence intervals will be reported for the total sample (by planned mode of cervical ripening – home or hospital, by actual mode (as some women who plan one mode may in practice have a different mode) and by study site. We will examine whether there are statistically significant differences in the primary outcome of sense of control (labour agentry) and by psychosocial outcome of postnatal psychological wellbeing score (WEMWBS) between women with home cervical ripening and women with in-hospital ripening. The covariates will

For peer review only

include the reason for IOL, gestational age, maternal age, parity, sociodemographic status, ethnic group.

The sample size required to compare the experiences of women who had home and hospital cervical ripening is estimated to be 46 subjects within each of 12 sites (assuming equal numbers within each site) i.e. 552 women in total. This is based on use of LAS(25) where a change of 5.5 points is considered clinically meaningful. In an individually randomised study, to have 90% power at a 5% level of significance to detect an effect size of 0.5 (two-sided) we would need 85 evaluable subjects per arm (170 total). However, this has to be inflated for the clustering within each site. We assume that the intraclass correlation coefficient is 0.05 in this setting. We will also inflate the target sample by 10% to account for incomplete data/ unusable questionnaires, aiming for 613 women in total.

We will invite all sites using accessible electronic maternity records to participate in the survey; but with the option to opt out. We will include at least 12 sites, i.e. a total of at least 43,200 births annually across the sites. Our previous experience of questionnaire-based surveys, and the UK's national maternity experience suggest a response rate of 40%. With an estimated eligibility of 22% of all maternities having IOL at 39 weeks or more, and 15% of these having home cervical ripening. We expect to achieve our sample size within four months and will monitor recruitment rates from each site and if necessary extend the survey period to ensure an adequate sample.

Case studies

In-depth case studies will be undertaken at five sites. The sample of five case studies is pragmatic, and selection is designed to balance depth with breadth of information and analysis with sites chosen to provide diversity and balance of service types on the basis of geography,

For peer review only

service configuration and approaches to provision of IOL. We will undertake semi-structured interviews with women, their partners and a range of staff and stakeholders in each site. A topic guide and pathway mapping will be used to help focus the discussion. Interviews will explore perceptions and experiences of the service approach to induction and implementation of local cervical ripening protocols in practice.

Women and partner interviews

Women will be eligible for interview if they have IOL at a gestation of 39^{+0} weeks gestation or more, have given birth in one of the case study sites and responded to the survey indicating a willingness to be contacted regarding the interview.

A purposive sample of women will be included. A sampling frame will be constructed within and across case study sites with the aim of including a balance of nulliparous and parous women, women who were offered outpatient cervical ripening but declined, women who experienced this and women who were not offered it. The women approached will be given the opportunity to ask further questions and at least one week to decide whether to participate in an interview. Interviews will be conducted online or by telephone using a verbal consent protocol prior to the start of the interview. All women who consent to participate in an interview will also be asked whether they give consent for their birth partner to be invited for an interview. We anticipate interviewing between 10-15 women in each site (total 50-75 participants) and, assuming that around half of participants may have a birth partner willing to participate, we anticipate including around 25-38 birth partners. If couples express a preference to be interviewed together, this will be accommodated. 'Birth partner' will be defined by the women themselves.

For peer review only

Key professionals, stakeholders and maternity professionals' interviews and focus groups

Key professionals and stakeholders for interviews will be identified with the support of the PI for each local case study service but will typically include: head of midwifery, clinical director, consultant obstetricians and midwives, chairs of local Maternity Voices Partnerships, representatives from local maternity service user groups and service commissioners or health board leads. Interviews will be conducted online or by telephone. Verbal consent will be obtained at the start of the interview. We anticipate undertaking around 10 individual interviews in each case study site.

Midwives and obstetricians will be invited to participate in focus group discussions and we estimate that three focus groups comprising of six to eight participants (total 18-24 participants) will be held in each site. These will be organised to facilitate participation of a diversity of maternity professionals, by including in a local audit meeting or study day. Focus groups may be held online or in person if access to the case study site is possible.

Observations of maternity visits discussing IOL

A small convenience sample of maternity visits will be included in each case study site in order to enable analysis of information provision and women's information needs. Up to five maternity professionals in each site will be provided with a digital recorder and given instructions on use and asked to record three consecutive interviews with the woman's consent. We will follow up the recorded consultations with a brief (up to ten minute) telephone interview to explore the woman's understanding of the information provided.

For peer review only

Qualitative data analysis

All qualitative data will be transcribed and entered into the analysis support software NVivo to support data management and analysis. Documentary sources will be added to the NVivo project file as PDF files. Visual approaches will be used to support the discussion and analysis of the pathways. Recordings of discussions will be analysed using a structured approach to conversation analysis. Interviews with women, partners and health professionals will be transcribed and analysed using a thematic framework approach, based on frameworks developed in recent work by the study team as part of the PROBIT-F trial (8, 26).

RESEARCH ETHICS APPROVAL AND DISSEMINATION

CHOICE has National Research Ethics Service Committee approval (York and Humber - Sheffield Research Ethics Committee, REC reference: 20/YH/0145), National R&D approval in Scotland (NHS Research Scotland Permissions) and England (Health Research Authority), and approval from the Public Benefit and Privacy Panel (PBPP) in Scotland is pending. The study will be conducted in accordance with the principles of the International Conference on Harmonisation Tripartite Guideline for Good Clinical Practice (ICH GCP).

CHOICE is registered on the ISRCTN registry (ISRCTN32652461)

Results will be submitted for peer reviewed academic publication and presented at international conferences. Meta-data produced in this study will also become available to Health Data Research UK (HDRUK) Gateway. Strengthening the Reporting of Observational Studies in Epidemiology (STROBE) guidance(16) and Reporting of studies Conducted using Observational Routinely-collected Data (RECORD) guidance(15) will be used to guide transparent reporting**.**

For peer review only

AUTHOR CONTRIBUTIONS

SJS, GS, JS and DP contributed to the conception of the study. All authors contributed to the study design. All authors contributed to drafting the protocol. SJS, HR and FW drafted the first version of the manuscript. All authors revised the manuscript for important intellectual content. All authors gave final approval of the version to be published

PATIENT AND PUBLIC INVOLVEMENT

There are two PPI co-applicants on the study grant who have contributed to the protocol, study design and patient facing materials. A CHOICE Parent Advisory Group (PAG) has been established to provide further support and guidance on study materials and management.

ACKNOWLEDGMENTS

We gratefully acknowledge the help of the following people:

Trial Steering Committee: Dr Brenda Kelly (Chair), Dr Sarah Stock, Fiona Fraser, Jaqueline Birks, Dr Tracey Mills, Dr Kate Walker, Fiona Wee.

The West Midlands NIHR Clinical Research Network, who are the lead network for the study in England, and are providing support with site feasibility and set up.

Clevermed Ltd, provider of BadgerNet maternity records system, who are providing technical expertise and facilitating data extracts from approved sites.

The Edinburgh Clinical Trials Unit (ECTU), who are providing trial management, data management and statistical support.

For peer review only

Delivered Social, who kindly created and donated the CHOICE study website completely free of charge as part of their social impact programme; Delivered Social Green.

FUNDING

CHOICE is funded by the National Institute of Healthcare Research Health Technology and Assessment (NIHR HTA) NIHR 127569. The views expressed are those of the authors and not necessarily those of the NIHR or the Department of Health and Social Care.

SJS is supported by Wellcome Trust (209560/Z/17/Z)

For peer review only

COMPETING INTERESTS

SS reports grants from NIHR, grants from Wellcome Trust, grants from Tommy's Charity, grants from MRC, during the conduct of the study.

AB reports a grant from UK National Institute of Health Research, Research for patient benefit program (NIHR RfPB) for Trans-cervical balloon catheter and its comparison to sustained release prostaglandin use for out -patient induction of labour in low-risk women: A feasibility study for a randomised controlled trial. (PB-PG-0815-20022), outside the submitted work.

MR is an employee of Clevermed Ltd.

KB reports a grant from the NIHR during conduct of study

JS reports grants from NIHR during conduct of the study

NM leads and directs NNRD

GS reports grants from NIHR HTA, during the conduct of the study; personal fees from GlaxoSmithKline Research and Development Limited, grants from Sera Prognostics Inc,

non-financial support from Illumina Inc, grants, personal fees and non-financial support from Roche Diagnostics Ltd, grants from Medical Research Council, grants from NIHR HTA, grants from Wellbeing of Women, grants from Wellcome Trust, grants from NIHR, outside the submitted work; In addition, Dr. Smith has a patent Named inventor on patent application for a novel predictive test for fetal growth restriction (FGR) pending.

JN reports grants from University of Edinburgh, during the conduct of the study; and JN declares membership of the following NIHR boards: CPR Decision Making Committee, HTA Commissioning Board, HTA Commissioning Sub-Board (EOI), HTA Funding Boards Policy Group, HTA General Board, HTA Post-Board funding teleconference, NIHR CTU Standing Advisory Committee, NIHR HTA and EME Editorial Board and Pre-exposure Prophylaxis Impact Review Panel; and the MRC/NIHR EME Funding Board.

DATA STATEMENT

Individual NHS organisations are the data controllers for the study. We will not be able to share data for the study as we are not the data controllers. All results put into the public domain will be subject to statistical disclosure control according to usual processes. Meta-data produced in this study will be made available to Health Data Research UK (HDRUK) Gateway. Applications to use the Scottish datasets included in the study can be made via https://www.informationgovernance.scot.nhs.uk/pbpphsc/

For peer review only

REFERENCES

1. National Maternity and Perinatal Audit: Clinical Report 2019. Based on births in NHS maternity services between 1 April 2016 and 31 March 2017. London: RCOG; 2019.

2. Grobman WA, Rice MM, Reddy UM, et al. Labor Induction versus Expectant Management in Low-Risk Nulliparous Women. N Engl J Med 2018;379(6):513-23;

3. Stock SJ, Ferguson E, Duffy A, et al. Outcomes of elective induction of labour compared with expectant management: population based study. BMJ. 2012;344:e2838.

4. Knight HE, Cromwell DA, Gurol-Urganci I, et al. Perinatal mortality associated with induction of labour versus expectant management in nulliparous women aged 35 years or over: An English national cohort study. PLoS Med. 2017;14(11):e1002425.

5. Royal College of Midwives. The Gathering Storm: England's Midwifery Workforce Challenges, 2017

6. Shetty A, Burt R, Rice P, et al. Women's perceptions, expectations and satisfaction with induced labour--a questionnaire-based study. Eur J Obstet Gynecol Reprod Biol. 2005;123(1):56-61.

7. Henderson J, Redshaw M. Women's experience of induction of labor: a mixed methods study. Acta Obstet Gynecol Scand. 2013;92(10):1159-67.

8. Coates R, Cupples G, Scamell A, et al. Women's experiences of induction of labour: qualitative systematic review and thematic synthesis. Midwifery. 2018.

9. Norman JE, Stock S. Intracervical Foley catheter for induction of labour. Lancet. 2011;378(9809):2054-5.

For peer review only

10. NICE Guideline CG70. Induction of labour 2008.

11. Jozwiak M, Oude Rengerink K, Benthem M, et al. Foley catheter versus vaginal prostaglandin E2 gel for induction of labour at term (PROBAAT trial): an open-label, randomised controlled trial. Lancet. 2011;378(9809):2095-103.

12. Alfirevic Z, Gyte GML, Nogueira Pileggi V, et al. Home versus in patient induction of labour for improving birth outcomes. Cochrane Database of Systemic Reviews 2020, Issue8. Art No.: CD007372.DOI:10.1002/14651858.CD007372.pub4

13. Cheyne H, McCourt C, Semple K. Mother knows best: developing a consumer led, evidence informed, research agenda for maternity care. Midwifery. 2013;29(6):705-12

14. Dos Santos F, Drymiotou S, Antequera A, et al. Development of a core outcome set for trials on induction of labour (COSIOL): An international multi-stakeholder Delphi study. BJOG. 2018.

15. Benchimol EI, Smeeth L, Guttmann A, et al; RECORD Working Committee. The REporting of studies Conducted using Observational Routinely-collected health Data (RECORD) statement. PLoS Med. 2015 Oct 6;12(10):e1001885. doi: 10.1371/journal.pmed.1001885. eCollection 2015 Oct.

16. von Elm E, Altman DG, Egger M, et al; STROBE Initiative. The Strengthening the Reporting of Observational Studies in Epidemiology (STROBE) statement: guidelines for reporting observational studies. PLoS Med. 2007 Oct 16;4(10):e296.

17. Jozwiak M, Oude Rengerink K, Ten Eikelder ML, et al. Foley catheter or prostaglandin E2 inserts for induction of labour at term: an open-label randomized controlled trial (PROBAAT-P trial) and systematic review of literature. Eur J Obstet Gynecol Reprod Biol. 2013;170(1):137-45.

For peer review only

18. Consolidated Health Economic Evaluation Reporting Standards (CHEERS) statement BMJ 2013; 346 doi: https://doi.org/10.1136/bmj.f1049

19. Unit Costs of Health and Social Care 2018: University of Kent at Canterbury: PSSRU, 2018

20. Kreif N, Grieve R, Sadique MZ. Statistical methods for cost-effectiveness analyses that use observational data: a critical appraisal tool and review of current practice. Health Econ. 2013;22(4):486-500

21. https://www.onlinesurveys.ac.uk Accessed December 2020

22. Vogel JP, Osoti AO, Kelly AJ, et al. Pharmacological and mechanical interventions for labour induction in outpatient settings. Cochrane Database Syst Rev. 2017;9:CD007701.

23. Adelson PL, Wedlock GR, Wilkinson CS, et al. A cost analysis of inpatient compared with outpatient prostaglandin E2 cervical priming for induction of labour: results from the OPRA trial. Aust Health Rev. 2013;37(4):467-73.

24. Sharp AN, Stock SJ, Alfirevic Z. Outpatient induction of labour in the UK: a survey of practice. Eur J Obstet Gynecol Reprod Biol. 2016;204:21-3.

25. Kelly AJ, Alfirevic Z, Ghosh A. Outpatient versus inpatient induction of labour for improving birth outcomes. Cochrane Database Syst Rev. 2013(11):CD007372.

For peer review only

26. Rose Coates, Georgina Cupples, Amanda Scamell, et al. Women's experiences of outpatient induction of labour with double balloon catheter or prostaglandin pessary: A qualitative study, Women and Birth (2020), https://doi.org/10.1016/j.wombi.2020.07.00

For peer review only

TABLES

Table 1: Secondary outcomes

**Safety outcomes**	***Baby***	***Maternal***
	Any neonatal unit admission (any level of care)	Intensive care unit transfer
	Neonatal intensive care unit (NICU) admission	High dependency level care
	Duration of neonatal unit stay	Hyperstimulation or tachysystole (as defined by care givers)
	Duration of NICU stay	Hyperstimulation or tachysystole causing CTG abnormality (as defined by care givers)
	APGAR score <7 at 5 minutes	Umbilical cord prolapse
	APGAR score <4 at 5 minutes	Birth outwith hospital
	Arterial Cord Blood pH <7.1	Postpartum haemorrhage 1000ml or more
	Arterial Cord base excess >12mmol/L	Maternal pyrexia 38 °C or more after commencing cervical ripening (*Exploratory outcome)*
	Neonatal Seizures	
	Hypoxic Ischaemic Encephalopathy (as recorded by care givers)	
	Level 2 or Level 3 Hypoxic Ischaemic Encephalopathy (as recorded by care givers)	

For peer review only

	Meconium aspiration syndrome<br><br>Mechanical ventilation<br><br>Intracranial haemorrhage<br><br>Stillbirth after admission/first attendance for induction of labour (excluding deaths from congenital anomalies)<br><br>Early neonatal death up to 7 days after birth (day 0-6; excluding deaths from congenital anomalies)<br><br>Treatment for neonatal sepsis [defined as positive blood, cerebral spinal fluid, or urine culture or cardiovascular collapse or X-ray confirming infection] *(Exploratory outcome)*<br><br>Treatment in neonatal unit for neonatal infection (defined as antibiotic treatment and Temperature ≥37.5 °C or <35.5 °C) *(Exploratory outcome)*<br><br>Treatment for neonatal jaundice [defined as peak total bilirubin of at least 15mg or the use of phototherapy] *(Exploratory outcome)*	
**Effectiveness outcomes**	Time from first cervical ripening agent to admission to labour ward/birth unit<br><br>Time from first cervical ripening agent to birth	

For peer review only

	More than one cervical ripening agent used<br><br>Duration of antenatal hospital stay for cervical ripening<br><br>Duration of labour ward admission until birth<br><br>Duration postnatal hospital stay (mother)<br><br>Total hospital stay<br><br>Hours spent at home<br><br>Oxytocin use<br><br>Mode of birth<br><br>Birth in obstetric unit<br><br>Birth in alongside midwifery unit (if available at that site)
**Mother baby outcomes**	Breastfeeding at discharge from maternity care<br><br>Skin to skin at birth
**Cost effectiveness**	***Primary outcomes***<br><br>Incremental cost per neonatal admissions avoided (home versus in-hospital)<br><br>Incremental quality adjusted life year (QALYs) (home versus in-hospital)

For peer review only

	Other (exploratory) economic outcomes<br><br>Incremental cost per hour prevented from hospital admission to delivery/birth<br><br>Incremental cost per neonatal admission avoided (home balloon catheter versus home dinoprostone)<br><br>Incremental cost per QALY (home balloon catheter versus home dinoprostone)<br><br>Incremental cost per hour prevented from hospital admission to delivery/birth (home balloon catheter versus home dinoprostone
**Outcomes to check comparability of groups/matching**	Birthweight<br><br>Birthweight centile<br><br>Small for gestational age (<10th centile for gestational age)<br><br>Large for gestational age (>90th centile for gestational age)

For peer review only

**qCHOICE process evaluation outcomes**	***Primary Outcome***<br><br>Sense of control (agentry) in labour<br><br>***Secondary Outcomes***<br><br>Women's satisfaction with IOL care<br><br>Women's postnatal psychological wellbeing<br><br>Women's overall evaluation of their labour and birth experience (qualitative analysis)<br><br>Costs incurred by the woman and family

For peer review only

Figure 1. Process evaluation logic model – V1 18/12/18

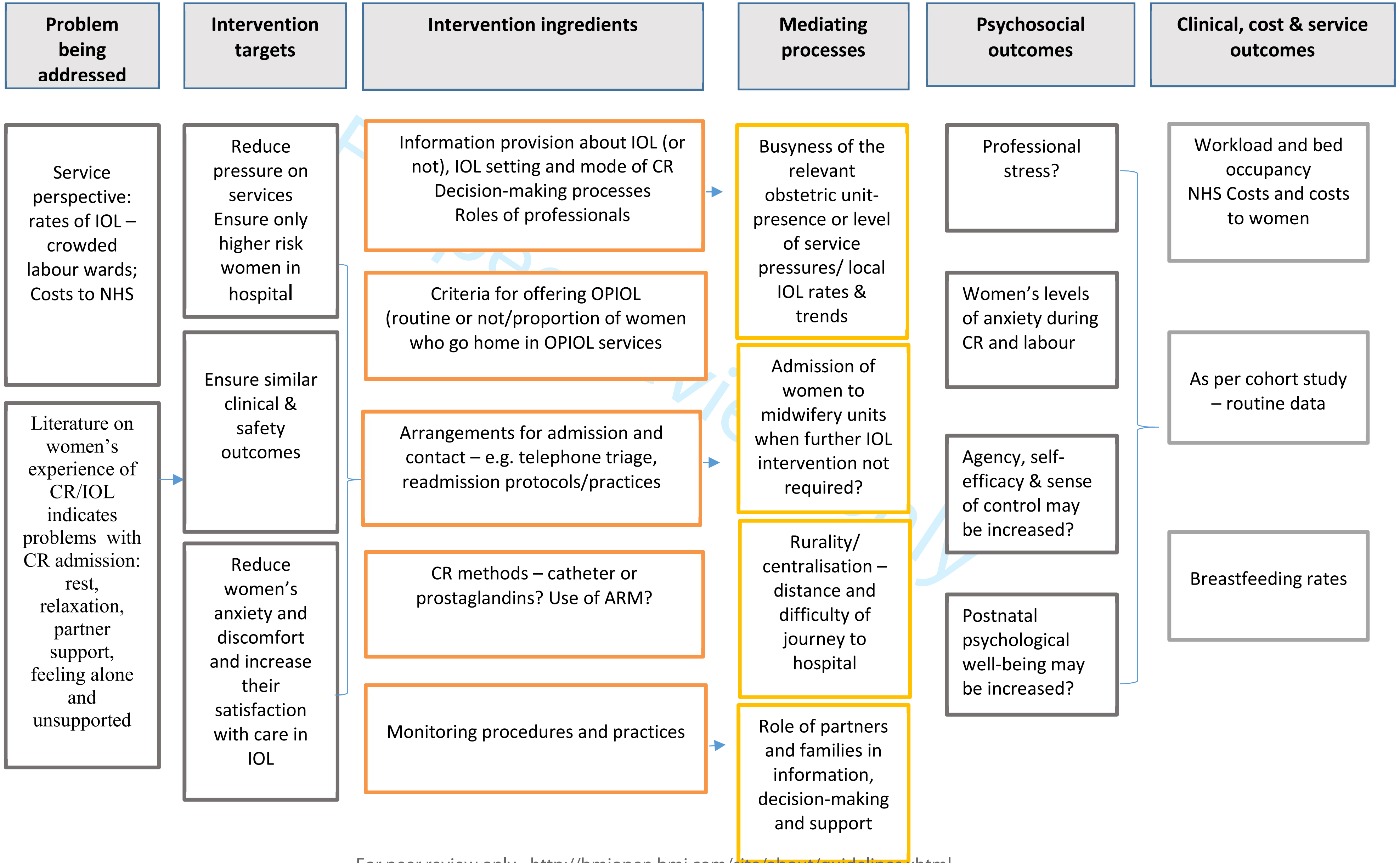

BMJ Open

Cervical ripening at home or in-hospital - prospective cohort study and process evaluation (CHOICE) study: Protocol

Journal:	*BMJ Open*
Manuscript ID	bmjopen-2021-050452.R1
Article Type:	Protocol
Date Submitted by the Author:	30-Mar-2021
Complete List of Authors:	Stock, Sarah; The University of Edinburgh<br>Bhide, Amarnath; St George's University of London<br>Richardson, Heather; NHS Lothian University Hospitals Division<br>Black, Mairead; University of Aberdeen<br>Yuill, Cassandra; City University of London<br>Harkness, Mairi; University of Stirling<br>Reid, Maggie; The University of Edinburgh<br>Wee, Fiona; The University of Edinburgh<br>Cheyne, Helen; University of Stirling<br>McCourt, Christine; City University, City University London<br>Rana, Dikshyanta; University of Glasgow, Institute of Health and Wellbeing<br>Boyd, Kathleen; University of Glasgow, Health Economics & Health Technology Assessment<br>Sanders, Julia; Cardiff University, School of Healthcare Sciences<br>Heera, Neelam; Patient and Public Involvement Representative<br>Huddleston, Jane; Patient and Public Involvement Representative<br>Denison, Fiona; The University of Edinburgh, MRC Centre for Reproductive Healht<br>Pasupathy, Dharmintra; The University of Sydney<br>Modi, Neena; Imperial College London, Medicine<br>Smith, Gordon; University of Cambridge, Obstetrics and Gynaecology<br>Norrie, John ; The University of Edinburgh
**Primary Subject Heading**:	Obstetrics and gynaecology
Secondary Subject Heading:	Obstetrics and gynaecology
Keywords:	Maternal medicine < OBSTETRICS, Fetal medicine < OBSTETRICS, OBSTETRICS

SCHOLARONE™
Manuscripts

I, the Submitting Author has the right to grant and does grant on behalf of all authors of the Work (as defined in the below author licence), an exclusive licence and/or a non-exclusive licence for contributions from authors who are: i) UK Crown employees; ii) where BMJ has agreed a CC-BY licence shall apply, and/or iii) in accordance with the terms applicable for US Federal Government officers or employees acting as part of their official duties; on a worldwide, perpetual, irrevocable, royalty-free basis to BMJ Publishing Group Ltd ("BMJ") its licensees and where the relevant Journal is co-owned by BMJ to the co-owners of the Journal, to publish the Work in this journal and any other BMJ products and to exploit all rights, as set out in our licence.

The Submitting Author accepts and understands that any supply made under these terms is made by BMJ to the Submitting Author unless you are acting as an employee on behalf of your employer or a postgraduate student of an affiliated institution which is paying any applicable article publishing charge ("APC") for Open Access articles. Where the Submitting Author wishes to make the Work available on an Open Access basis (and intends to pay the relevant APC), the terms of reuse of such Open Access shall be governed by a Creative Commons licence – details of these licences and which Creative Commons licence will apply to this Work are set out in our licence referred to above.

Other than as permitted in any relevant BMJ Author's Self Archiving Policies, I confirm this Work has not been accepted for publication elsewhere, is not being considered for publication elsewhere and does not duplicate material already published. I confirm all authors consent to publication of this Work and authorise the granting of this licence.

For peer review only

Cervical ripening at home or in-hospital - prospective cohort study and process evaluation (CHOICE) study: Protocol

Sarah J Stock, Usher Institute, University of Edinburgh NINE Edinburgh BioQuarter, 9 Little France Road, Edinburgh EH16 4UX UK sarah.stock@ed.ac.uk +44 (0)7894629934 (corresponding author) ORCID ID 0000-0003-4308-856X

Dr Amarnath Bhide, Consultant Obstetrician, Obstetrics and Gynaecology, St George's University Hospitals Trust, London, UK. ORCID ID: 0000-3-2393-7501

Heather Richardson, Subspecialty Registrar in Maternal and Fetal Medicine, Simpson Centre for Reproductive Health, NHS Lothian, Edinburgh, UK

Dr Mairead Black, Senior Clinical Lecturer, Institute of Applied Health Sciences, University of Aberdeen, Aberdeen, UK

Dr Cassandra Yuill, Research Fellow, Centre for Maternal & Child Health Research School of Health Sciences, City, University of London, London, UK

Dr Mairi Harkness, Nursing, Midwifery and Allied Health Professions Research Unit (NMAHP), Research Fellow, University of Stirling, Stirling, UK

Mrs Maggie Reid, Research Midwife, Usher Institute, University of Edinburgh, Edinburgh, UK

For peer review only

Mrs Fiona Wee, Trial Manager, Usher Institute, University of Edinburgh, Edinburgh, UK

Professor Helen Cheyne, Royal College of Midwives Professor of Midwifery, Nursing Midwifery and Allied Health Professions Research Unit (NMAHP), University of Stirling, Stirling, UK. ORCID ID: 0000-0001-5738-8390

Professor Christine McCourt, Professor of Maternal and Child Health, School of Health Sciences, City, University of London, London, UK

Miss Dikshyanta Rana, Research Assistant, Institute of Health and Well Being, University of Glasgow, Glasgow, UK

Dr Kathleen Boyd, Senior Lecturer, Institute of Health and Well Being, University of Glasgow, Glasgow, UK

Professor Julia Sanders, Professor of Clinical Nursing & Midwifery, School of Healthcare Sciences, Cardiff University, Cardiff, UK

Miss Neelam Heera, Patient and Public Involvement Representative

Mrs Jane Huddleston, Patient and Public Involvement Representative

Professor Fiona Denison, Reader/Honorary Consultant in Maternal and Fetal Medicine, MRC Centre for Reproductive Health, University of Edinburgh, Edinburgh, UK

For peer review only

Professor Dharmintra Pasupathy, Professor of Maternal Fetal Medicine, Faculty of Medicine and Health, University of Sydney, Sydney, Australia

Professor Neena Modi, Professor of Neonatal Medicine, School of Public Health, Imperial College London, London, UK

Professor Gordon C S Smith, Professor of Obstetrics and Gynaecology, Department of Obstetrics and Gynaecology, The University of Cambridge, Cambridge, UK

Professor John Norrie, Professor of Medical Statistics and Trial Methodology / Director of Edinburgh Clinical Trials Unit / Co-Head of Centre for Population Health Sciences, Usher Institute, University of Edinburgh, Edinburgh, UK

Word count: 6222

Key words: Induction of labour, Prostaglandin, Cervical Ripening Balloon, Pregnancy

For peer review only

ABSTRACT

Introduction

The aim of the CHOICE study is to compare home versus in-hospital cervical ripening to determine; whether home cervical ripening is safe (for the primary outcome of neonatal unit [NNU] admission), acceptable to women, and cost-effective from the perspective of both women and the NHS.

Methods and analysis

We will perform a prospective multicentre observational cohort study with an internal pilot phase. We will obtain data from electronic health records from at least 14 maternity units offering only in-hospital cervical ripening and 12 offering dinoprostone home cervical ripening. We will also conduct a cost-effectiveness analysis and a mixed methods study to evaluate processes and women/partner experiences. Our primary sample size is 8,533 women with singleton pregnancies undergoing induction of labour (IOL) at 39^{+0} weeks' gestation or more. To achieve this and contextualise our findings, we will collect data relating to a cohort of approximately 41,000 women undergoing IOL after 37 weeks.

We will use mixed effects logistic regression for the non-inferiority comparison of neonatal unit admission and propensity score matched adjustment to control for treatment indication bias.

The economic analysis will be undertaken from the perspective of the National Health and Personal Social Services (NHS and PSS) and the pregnant woman. It will include a within-study cost-effectiveness analysis and a lifetime cost-utility analysis to account for any long-term impacts of the cervical ripening strategies. Outcomes will be reported as incremental cost

For peer review only

per NNU admission avoided and incremental cost per quality adjusted life year (QALY) gained.

Research Ethics approval and Dissemination

CHOICE has been funded and approved by the National Institute of Healthcare Research Health Technology and Assessment (NIHR HTA), and the results will be disseminated via publication in peer reviewed journals.

ARTICLE SUMMARY

Strengths and limitations of this study

- This is a large study to evaluate the safety of at-home cervical ripening
- We will set up a platform for data collection using electronic health records to enable future research into rare safety outcomes
- The study includes assessment of women's views and experiences using validated questionnaires as well as qualitative methods.
- Observational design of the study makes it vulnerable to residual confounding.

For peer review only

INTRODUCTION

Induction of labour (IOL) is the most common obstetric intervention offered to women when risks of continuing the pregnancy are thought to outweigh risks of birth. Increases in IOL rates over the past 10 years mean that now 29.6% of all pregnant women in the UK have their labour induced.(1) IOL at term, when compared to expectant management of pregnancy, reduces caesarean birth, maternal hypertensive disease and complications,(2) as well as being associated with a reduction in perinatal mortality.(3, 4) The demands on maternity services are increasing to accommodate increasing rates of IOL.(5) Although IOL (compared to expectant management) reduces overall hospital stay, it increases the time on labour and delivery wards,(2) having a major impact on resources, staffing, and negatively impacts on women's experience of labour.(6-8)

Cervical ripening is a key component of IOL.(9) It may initiate labour, but is often followed by artificial rupture of membranes +/- intravenous oxytocin infusion. NICE guidance(10) recommends pre-induction cervical ripening in all women having IOL unless there is a contraindication.

Traditionally cervical ripening has been performed entirely in-hospital, to allow monitoring of maternal/fetal wellbeing and early recognition of complications.(11) However, an increasing number of UK maternity units offer outpatient (home) cervical ripening. As the rate of IOL is increasing, home cervical ripening may provide opportunities to reduce the burden on the NHS, for example, by reducing hospital stay during IOL. However, the safety and acceptability of home cervical ripening have not been fully evaluated. NICE(10) identified the need to assess the safety, efficacy and clinical and cost-effectiveness of outpatient and inpatient IOL in the UK setting, taking into account women's views. A recent Cochrane review found insufficient evidence to draw conclusions on the efficacy, safety and cost-effectiveness of home-induction

For peer review only

of labour and indicated that large prospective cohort studies would be needed.(12) Maternity service users have identified IOL as an important research topic,(13) and women have reported specific negative experiences such as increased pain and anxiety and lack of support which may be alleviated by home cervical ripening.(8) Potential NHS cost savings of home cervical ripening could be offset by increased costs of any additional morbidity resulting from home cervical ripening, costs to parents may be increased, and acceptability of home cervical ripening is unknown. Health services need to balance the full resource impact of IOL with the need to provide safe and acceptable care.

In the cervical ripening at home or in-hospital – prospective cohort study and process evaluation (CHOICE) study, we will perform an observational cohort study with a cost-effectiveness analysis and process evaluation to address the question "Is it safe, effective, cost-effective and acceptable to women to carry out home cervical ripening during IOL?" These analyses will provide information to help women and their caregivers make informed decisions around how to have IOL.

Our main aim is to compare the setting of cervical ripening at home versus in-hospital. As the NICE recommended agent for cervical ripening is vaginal prostaglandin, our primary comparison will be home dinoprostone versus in-hospital dinoprostone. In order to future-proof the study, we will include a secondary comparison: home cervical ripening with balloon catheter versus home cervical ripening with dinoprostone. By including two different methods of home cervical ripening within our study, we will provide initial comparative evidence on these two methods of home labour induction.

For peer review only

METHODS AND ANALYSIS

CHOICE Prospective Cohort study

Study design and setting

We will carry out a prospective multicentre cohort study using de-identified clinical data from electronic hospital records. The primary outcome will be non-inferiority of NNU/special care admission for 48 hours or longer, initiated within 48 hours of birth.

The study will be performed in at least 26 UK obstetric units, 14 of which offer exclusively in-hospital cervical ripening and 12 offer dinoprostone cervical ripening both in-hospital and at home.

Participating maternity units will be purposively selected to represent the diverse range of maternity service settings in the UK, and include urban tertiary referral units, mid-sized urban district general hospitals and small, more isolated, rural units.

Data sources

Data will be collected directly from electronic maternity (and neonatal) records for participants who had babies admitted to a neonatal unit. These data are recorded by clinical staff (midwives, doctors and neonatal nurses) during the course of antenatal, intrapartum and postpartum care. Existing data fields, supplemented by new bespoke, data entry fields enabled in the maternity dataset at participating sites will be used. Unless women opt-out of secondary data use (from similar studies we estimate <1% will opt out), de-identified data will be transferred from participating sites to a secure University of Edinburgh server for analysis.

For peer review only

No personal data will be collected. Potentially identifiable data, such as the date and time of birth, date of events such as commencing cervical ripening, hospital discharge, will be converted into gestation at birth (weeks + days); and antenatal and postnatal events into "t–x" and "t+x" hours and days respectively.

Population, inclusion and exclusion criteria

We will initially apply broad inclusion criteria and collect data from all women having IOL at 37^{+0} weeks gestation or more to create a cohort for analyses. Women who have opted out of data provision will be excluded.

We will then apply more stringent inclusion and exclusion criteria at the analysis stage for a suite of nested analyses. In our primary analysis, we will create a cohort of women with "uncomplicated" (ie those with no identified risk factors for adverse maternal or perinatal outcomes defined below) pregnancies in whom there was no contraindication to home cervical ripening, who had singleton pregnancies with IOL at 39 weeks gestation or more. This group will include women having IOL for post-dates, but also women having IOL because of maternal or clinician preference, IOL for maternal age, IOL for discomfort or social indications. Exclusion criteria will consist of grand multiparity (6 or more previous births), previous caesarean section, antepartum stillbirth (before cervical ripening initiated), Class III obesity at booking (BMI 40 kg/m^2 or more), Prelabour rupture of membranes (ROM) documented as primary or other indication for IOL (prolonged ROM; Spontaneous ROM; Suspected Spontaneous ROM), Maternal or fetal condition that would or could preclude home cervical ripening documented as primary or other indication for IOL (Maternal conditions: proteinuria; hypertension; antepartum haemorrhage; diabetes; obstetric cholestasis; past obstetric history; pre-eclampsia; PIH/PET (not defined); PIH; PET; thrombophilia. Fetal conditions: oligohydramnios; reduced liquor volume; macrosomia;

intrauterine growth restriction (IUGR); static growth; congenital fetal anomaly; polyhydramnios; abnormal CTG/Doppler; breech; reduced fetal movements; termination of pregnancy for fetal anomaly).

We will explore the potential for additional analyses which may include IOL for other indications (e.g. reduced fetal movements) or in other populations (e.g. multiple pregnancies; women with a previous caesarean birth). However, in general, home cervical ripening is only offered to 'low risk' women, so we anticipate that numbers of higher risk women having home IOL may not be high enough for meaningful analyses.

Exposure and Outcomes

Our primary aim is to compare home versus in-hospital cervical ripening. We will collect data at individual-level. The exposure group will be women who, at the start of the cervical ripening process, plan to have home cervical ripening. The comparator group will be women who planned to have in-hospital cervical ripening from maternity units not offering home cervical ripening. This will minimise potential bias arising from the fact that, in maternity units which offer both home and in-hospital cervical ripening, the risk of complications in the babies of women having home cervical ripening (lower risk pregnancies) is inherently different to that of babies of women having in-hospital cervical ripening (higher risk pregnancies).

As the NICE recommended agent for cervical ripening is vaginal prostaglandin, our primary comparison will be home dinoprostone versus in-hospital dinoprostone. Dinoprostone is now most commonly administered as 10mg slow-release pessary (Propess, Ferring) which stays in place for 24 hours. We will use this formulation in our primary comparison.

We will include a secondary exploratory comparison - home cervical ripening with balloon catheter (exposure) versus home cervical ripening with dinoprostone (comparator), to explore

For peer review only

if there are any indications of different safety profiles of these two methods of home cervical ripening.

Our proposed primary outcome will be admission to a NNU/Special care baby unit for 48 hours or longer, initiated within 48 hours of birth. NNU admission is a marker of neonatal morbidity and is the leading core outcome defined for studies of IOL.(14) Any increase in NNU admission of term babies is undesirable due to the separation of mother and baby. However, NNU admission rates are highly variable between maternity units and are likely to depend on local policies and culture. We therefore, plan to use a primary outcome which represents more severe neonatal morbidity (admission to a NNU within 48 h of birth for 48 h or longer) which is less likely to be influenced by site-specific factors. We may re-define the parameters of NNU admission used in the primary outcome after analysis of pilot data (see section on ‘Pilot phase’).

We have prespecified a number of secondary outcomes to assess the safety of home cervical ripening with respect to neonatal and maternal morbidity, shown in Table 1. These include outcomes from a core outcome set for studies of IOL.(14) We will also include secondary outcomes relating to the effectiveness of home cervical ripening, to explore whether the setting of cervical ripening influences subsequent labour and birth. Mother and baby outcomes were suggested by our lay consultation as important to include. We will use birthweight, birthweight centile, small for gestational age and large for gestational age as parameters to check the validity of our matching procedures in analyses. Birthweight is an objective outcome that may represent pregnancy complications, but extremely unlikely to be affected by the setting of cervical ripening. Comparison of birthweights between groups should provide reassurance that we have minimised systemic bias in our analyses.

For peer review only

Statistical analysis

All analyses will be fully specified in a comprehensive Statistical Analysis Plan and agreed by the Steering Committee. Analyses will be carried out in accordance with relevant guidance including RECORD(15) and STROBE.(16)

We will include at least 14 maternity units offering only in-hospital cervical ripening and 12 offering dinoprostone home cervical ripening (~95,000 deliveries per annum). We will invite additional maternity units to opt in to data provision, to allow contingency in case of 'cross overs' due to sites changing their IOL protocols during the study period.

We considered a superiority design for CHOICE, but decided against it because i) safety is a key concern to both clinicians and women, and was specified as the important outcome in the commissioning brief; ii) it is not plausible to hypothesise that home cervical ripening (intervention) is safer than in-hospital cervical ripening (comparator – the standard of care); and iii) it is not ethical to use a superiority design to test an intervention which may be worse (in terms of safety) than the established standard. Therefore, a non-inferiority design was chosen with a non-inferiority margin of 4% (deemed as likely to be an important difference on consultation with women and clinicians) for the primary outcome of neonatal unit admission.

Establishing the appropriate non-inferiority margin was complicated by recognition that the dimensions that are hypothesised to show benefit, i.e. acceptability to women and partners, and a reduction in costs appeal to different audiences – women will be primarily interested in acceptability and largely indifferent to costs (in a free at point of care NHS), whereas the potential reduction in costs will likely be the primary focus for the healthcare provider. We were also conscious that due to the inflation of the sample size due to (a) clustering; (b) losses due to non-matching in the propensity analysis and (c) loss to follow up, the sample size for a

For peer review only

smaller non-inferiority margin would quickly become not feasible within a realistic budget and timeframe. However, given that, regardless of a superiority or non-inferiority design, any specific sample size will estimate the treatment effect to a certain level of precision (e.g. the width of a 95% confidence interval), we are confident that our final comparison group of 1,920 in each arm (with ~ 115 NNU admissions in each arm [see sample size calculation below]), we will generate sufficient high-quality evidence to definitively answer the questions around safety, effectiveness, acceptability, and cost-effectiveness for this important question

For the principal analysis of the primary outcome, we will use mixed effects logistic regression for the non-inferiority comparison of NNU admission within 48 hours of birth for 48 hours or longer (Yes/No). As sensitivity analyses to demonstrate that the estimated treatment effects are robust to the chosen method, we will also explore propensity score weighting (PSW by inverse probability of receiving specified treatment) and single-stage regression, without using any propensity scoring, adjusting directly for the baseline factors relevant for treatment indication. We will also use propensity score matched (PSM) adjustment to control for treatment indication bias. The logistic model underlying the PSM will include variables such as age, Bishop's score, previous vaginal birth, co-morbidities, and relevant hospital-level factors, with 1:1 matching. Potential confounding variables will be identified before the start of the analysis, and these will be finalised after exploration of the data at the pilot stage, through the creation of directed acyclical graphs.

Similar analyses will be used for analyses of secondary outcomes, using logistic, linear, negative binomial, and time-to-event regressions. For example, we will analyse the duration of hospital stay during IOL, time spent at home, total hospital stay, and time to birth using linear models; while birth outwith hospital and breastfeeding will be analysed using logistic regression; and mode of birth using multinomial logistic regression.

For peer review only

For the remaining maternal secondary outcomes, we will include hyperstimulation, ≥1 induction agent, oxytocin use for induction or augmentation, maternal ICU/HDU admission, haemorrhage, uterine rupture, pulmonary embolus, and cardio-respiratory arrest. For the neonatal secondary outcomes, we will include meconium aspiration syndrome, respiratory support, neonatal infection, umbilical cord prolapse, neonatal birth trauma, neonatal encephalopathy (Grade II/III), therapeutic hypothermia and neonatal death. Logistic regression and Poisson or negative binomial regression, possibly inflated for excess zeros will be used as appropriate. For outcomes with a small number of events, we will use the appropriate exact regression procedure. As per the primary outcome, we will assess the influence of missing data for secondary outcomes using appropriate sensitivity-type analyses. We recognise that there are many secondary outcomes being analysed, as per the recommended core outcome set (17). We do not propose to make any formal statistical adjustment for the multiple comparisons. However, a caveat will be clearly expressed regarding the possibility of type 1 statistical error, given the multiple comparisons made. We will consider the following subgroup analyses, based on sufficient numbers to allow meaningful analyses: 1) nulliparous and parous women; 2) indication for IOL (post-dates IOL; maternal or clinician preference; maternal age; discomfort or social indication)

We propose the following sensitivity analyses. 1) Within-site comparison of home versus in-hospital cervical ripening (restricted to sites that offer home cervical ripening) 2) Per protocol analysis (women who actually are discharged home after commencing cervical ripening) 3) Complete case analysis to assess the effect of any strategies to deal with missing data.

Data from the larger cohort of women having IOL at 37^{+0} weeks' gestation or more, will be used to contextualise our findings on the background of unit practices and populations undergoing IOL. There is considerable inter-unit variation in both the rates of IOL and the risk

For peer review only

profile of women giving birth, that need to be considered. It will also allow us to capture any changes in practice over the study period regarding criteria for eligibility for home cervical ripening and change in method of IOL. This will help ensure the generalisability of our findings.

Future long-term outcome evaluation will be possible through data linkage to Hospital Episode Statistics and Scottish Morbidity Records.

Missing data

We anticipate missing data, but estimate that no more than 10% of women will have missing usable data on primary outcome, eligibility, setting of cervical ripening and/or have some part of the baseline data (age, co-morbidities, and any relevant identified hospital-level factors). We will use evidence-based strategies to minimise any such losses and recover any missing data that is possible. We will monitor levels of missing data as the study progresses, identifying any outcomes or exposures and/or sites that are prone to missingness, and take corrective action (e.g. additional feedback and support). We will conduct appropriate sensitivity type analyses, for example, using a multiple imputation approach assuming data are missing at random; and, if the data warrant (for example, if there is differential missingness between the in-hospital and at-home cohorts) non-ignorable (informative) missing data generating mechanisms.

We will also conduct an exploratory analysis comparing the two methods of home IOL, i.e. dinoprostone vs. mechanical methods. We will use the same methods as outlined above for the primary and secondary outcomes in the overall analysis.

For peer review only

Sample size

The sample size is based on our principal analysis (women with singleton pregnancies having IOL at 39 weeks gestation or more) and primary comparison (home cervical ripening vs in-hospital cervical ripening with dinoprostone), estimated 6% NNU admission rate (1) for babies born to mothers having IOL at >39 weeks gestation with no more than 4% excess NNU admission rate (from 6%), at 90% power, 2.5% 1-sided alpha, and an estimated ICC of 0.01. We will require 160 women in each of 12 sites (clusters) with uncomplicated pregnancies at 39 weeks or more undergoing IOL (total 1,920 in each arm). To account for the fact that i) only around 50% of women eligible for home cervical ripening in the intervention arm will actually initiate home cervical ripening, and ii) a larger pool of women is required in the control arm to allow for propensity score matching, our required sample size is 1,920 *2 (number of arms) / 0.5 (numbers of women actually starting home cervical ripening and matching) / 0.9 (for missing data), giving an overall required sample size of 8,533.

Based on an estimate that 22% of all maternities have IOL at 39 weeks or more(1) and that ~29% of these would be eligible for participation in our principal analysis (from scoping data from potential participating sites), and, in home cervical ripening sites ~50% of these will take up home cervical ripening, we anticipate achieving our recruitment targets within 20 months.

Current data from the NMPA for 2019 suggests that the national average rate of IOL after 37 weeks is 29.6%.(1) As our proposed participating units have about 90,000 births per annum, we anticipate collecting data on approximately 41,000 women having IOL at 37 weeks gestation or more.

For peer review only

Participant identification and opt out

Participants will be identified from data recorded in specified fields in electronic maternity records. We will use data fields indicating IOL, estimated due date (EDD) and date of IOL to identify women having IOL at 37 weeks gestation or more.

Women will be made aware of the CHOICE study through posters in participating sites; business cards; information leaflets; online adverts on hospital/maternity websites and relevant social media sites; and information in maternal electronic maternity records.

Women will be able to opt out of data provision by notifying their clinician or midwife, or emailing the study research midwife at the local site, and it will be recorded on their electronic record. There will be no restriction on co-enrolment in other studies.

Pilot phase

We propose a pilot phase to determine the parameters of the primary outcome and feasibility of obtaining the required sample size for analysis. This is based on the evaluable comparison group of 1,920 women in each arm, so acts as an inherent check on home cervical ripening eligibility and uptake rates, the assumed level of missingness and attrition due to non-matching. We will assess variation of the primary outcome at the pilot stage; along with that of other measures of neonatal morbidity included as secondary outcomes (e.g. any NNU admission, NICU admission). We may redefine the parameters of NNU admission used in the primary outcome after analysis of pilot data, choosing the one with the lowest ICC, or the one representing the least severe outcome which has an ICC of 0.01 or less. This decision will be made in consultation between the expert project management group, the trial steering committee (TSC) and the funder.

For peer review only

CHOICE Health Economic Analyses

Health economic analysis will be specified in a health economic analysis plan and reported in line with CHEERS guidelines.(18)

Economic analyses will explore the cost-effectiveness of at home versus inpatient cervical ripening for women undergoing IOL. Two separate cost-effectiveness questions will be addressed: (i) home cervical ripening with dinoprostone compared to in-hospital cervical ripening with dinoprostone and (ii) home cervical ripening with balloon catheter compared to home cervical ripening with dinoprostone. The evaluation will involve within study cost-effectiveness analysis and a lifetime cost-utility analysis to account for any long-term impacts (cost and morbidity) of the alternative cervical ripening strategies. Resource use data will be obtained from the prospective multicentre observational cohort study using data obtained from the maternity information system, Badgernet Maternity and National Neonatal Research Database (NNRD) data.

Costs incurred by women and their families relating to IOL are relevant from the patient perspective and potentially important for the 'at-home' cervical ripening strategy. These data are not available from the observational datasets, and therefore tailored economic-related questions have been incorporated into a process evaluation survey described in section 5 below.

To account for bias in the observational data, methods such as multivariate regression and propensity scoring will be employed as recommended in guidelines for cost-effectiveness analysis based on observational data,(19,20) which is consistent with the main study statistical analyses for this study. To capture any cost and morbidity events incurred in the neonatal period, the within-study analysis will include the primary study endpoint (NNU admission within 48 hours of birth for 48 hours or more) up to one-month post-birth. Outcomes will be

For peer review only

reported as the incremental cost per NNU admission avoided (in line with primary study outcome) as well as incremental cost per birth up to 28 days post-birth.

The lifetime analysis will account for longer term costs, quality of life, morbidity and disability from both the NHS & PSS and patient perspective and will report outcomes in terms of incremental cost per Quality Adjusted Life Years (QALYs) gained.

Qualitative (q)CHOICE Process evaluation

The process evaluation, nested within the observational cohort study, will comprise of a questionnaire-based survey in at least 12 sites and five case studies. Both qualitative and quantitative data will be collected, specifically a women's experience questionnaire, semi-structured interviews with women and birth partners, audio recordings of clinician/women consultations, interviews and focus-group discussions with professionals. Figure 1 describes the initial process evaluation logic model hypothesising the chain linking interventions and outcomes. This will inform data collection and analysis. At the final stage of data analysis, we will share and discuss emerging findings with a group of service users to develop a revised logic model and explanatory framework.

Questionnaire-based survey

Questionnaire data collection will take place over a four- to six-month period early in the study. CHOICE participating sites who use electronic maternity records accessible by women (the electronic equivalent of maternity hand-held records), will be invited to contribute to this part of the study. Women who have IOL at 39 weeks or more will receive a 'push or SMS notification' directing them to online study information when IOL is booked and a second notification around 10 days after they give birth. This will provide a link to the participant information sheets, consent form and online survey.

For peer review only

Push notifications are used by maternity services to prompt women to read information relating to their maternity care, including information within their electronic record. Women are able to opt out of SMS and push notifications, but routine monitoring of women's use of their online record shows that a sufficient number of women use notifications and continue to access their record postnatally, thus enabling a broad sample to be reached.

The survey landing page will include a summary of qCHOICE and links to the information sheets before directing women to the consent questions, which they are asked to complete before completing the survey. A telephone number will be supplied for women to call if they have any questions about the survey, or to request a postal survey if preferred. Surveys will be submitted online via Online Surveys,(21) by post or completed by phone with a member of the study team, with the support of an interpreter if needed. Participant contact details provided by survey respondents who are happy to be contacted further about a possible interview will be on a detachable back sheet of the questionnaire or a separate online page. Respondents will be informed that a £10 voucher will be offered to interview participants and that (with their consent) their birth partners may take part in an interview.

The survey will comprise validated tools as well as questions relating to service user costs, and the process of IOL as follows:

1. The Labour Agentry Scale (short form).(22) The LAS is a well-established, validated measure of women's experience during labour and birth. The short form LAS includes 10 items with a 7-point Likert type response. It measures perceived control during labour, which is the woman's sense of mastery over internal and environmental factors and is highly correlated with satisfaction with care. The LAS will be the primary outcome.

For peer review only

2. A modified version of the IOL satisfaction questionnaire(23) tested in the PROBIT-F trial. This questionnaire focuses specifically on women's experiences of aspects of IOL including information, anxiety and physical and emotional discomfort.

3. The short form Warwick-Edinburgh mental wellbeing scale WEMWBS.(24) A seven-item scale that measures mental wellbeing (as opposed to mental illness or disorder) representing positive attributes of wellbeing including feeling and functioning.

4. Additional questions which will inform the economic analysis from the woman's perspective will cover resource use and expenditures of cervical ripening for women including number of returns and phone calls to hospital, time distance, mode of travel to and from hospital, partner role, additional expenditure on maternity items and medication while at home, and additional childcare expenditure (if any) while at home.

5. The survey will include demographic questions, questions about the process of IOL, questions relating to the impact of COVID 19 on their experience of IOL, a question asking women if they would be willing to be contacted regarding possible participation in a semi-structured interview and for permission for data linkage to the observational cohort study.

Survey data will be analysed using descriptive and inferential statistics. Descriptive statistics with 95% confidence intervals will be reported for the total sample (by planned mode of cervical ripening – home or hospital, by actual mode (as some women who plan one mode may in practice have a different mode) and by study site. We will examine whether there are statistically significant differences in the primary outcome of sense of control (labour agentry) and by psychosocial outcome of postnatal psychological wellbeing score (WEMWBS) between women with home cervical ripening and women with in-hospital ripening. The covariates will

For peer review only

include the reason for IOL, gestational age, maternal age, parity, sociodemographic status, ethnic group.

The sample size required to compare the experiences of women who had home and hospital cervical ripening is estimated to be 46 subjects within each of 12 sites (assuming equal numbers within each site) i.e. 552 women in total. This is based on use of LAS(25) where a change of 5.5 points is considered clinically meaningful. In an individually randomised study, to have 90% power at a 5% level of significance to detect an effect size of 0.5 (two-sided) we would need 85 evaluable subjects per arm (170 total). However, this has to be inflated for the clustering within each site. We assume that the intraclass correlation coefficient is 0.05 in this setting. We will also inflate the target sample by 10% to account for incomplete data/ unusable questionnaires, aiming for 613 women in total.

We will invite all sites using accessible electronic maternity records to participate in the survey; but with the option to opt out. We will include at least 12 sites, i.e. a total of at least 43,200 births annually across the sites. Our previous experience of questionnaire-based surveys, and the UK's national maternity experience suggest a response rate of 40%. With an estimated eligibility of 22% of all maternities having IOL at 39 weeks or more, and 15% of these having home cervical ripening. We expect to achieve our sample size within four months and will monitor recruitment rates from each site and if necessary extend the survey period to ensure an adequate sample.

Case studies

In-depth case studies will be undertaken at five sites. The sample of five case studies is pragmatic, and selection is designed to balance depth with breadth of information and analysis with sites chosen to provide diversity and balance of service types on the basis of geography,

For peer review only

service configuration and approaches to provision of IOL. We will undertake semi-structured interviews with women, their partners and a range of staff and stakeholders in each site. A topic guide and pathway mapping will be used to help focus the discussion. Interviews will explore perceptions and experiences of the service approach to induction and implementation of local cervical ripening protocols in practice.

Women and partner interviews

Women will be eligible for interview if they have IOL at a gestation of 39^{+0} weeks gestation or more, have given birth in one of the case study sites and responded to the survey indicating a willingness to be contacted regarding the interview.

A purposive sample of women will be included. A sampling frame will be constructed within and across case study sites with the aim of including a balance of nulliparous and parous women, women who were offered outpatient cervical ripening but declined, women who experienced this and women who were not offered it. The women approached will be given the opportunity to ask further questions and at least one week to decide whether to participate in an interview. Interviews will be conducted online or by telephone using a verbal consent protocol prior to the start of the interview. All women who consent to participate in an interview will also be asked whether they give consent for their birth partner to be invited for an interview. We anticipate interviewing between 10-15 women in each site (total 50-75 participants) and, assuming that around half of participants may have a birth partner willing to participate, we anticipate including around 25-38 birth partners. If couples express a preference to be interviewed together, this will be accommodated. 'Birth partner' will be defined by the women themselves.

For peer review only

Key professionals, stakeholders and maternity professionals' interviews and focus groups

Key professionals and stakeholders for interviews will be identified with the support of the PI for each local case study service but will typically include: head of midwifery, clinical director, consultant obstetricians and midwives, chairs of local Maternity Voices Partnerships, representatives from local maternity service user groups and service commissioners or health board leads. Interviews will be conducted online or by telephone. Verbal consent will be obtained at the start of the interview. We anticipate undertaking around 10 individual interviews in each case study site.

Midwives and obstetricians will be invited to participate in focus group discussions and we estimate that three focus groups comprising of six to eight participants (total 18-24 participants) will be held in each site. These will be organised to facilitate participation of a diversity of maternity professionals, by including in a local audit meeting or study day. Focus groups may be held online or in person if access to the case study site is possible.

Observations of maternity visits discussing IOL

A small convenience sample of maternity visits will be included in each case study site in order to enable analysis of information provision and women's information needs. Up to five maternity professionals in each site will be provided with a digital recorder and given instructions on use and asked to record three consecutive interviews with the woman's consent. We will follow up the recorded consultations with a brief (up to ten minute) telephone interview to explore the woman's understanding of the information provided.

For peer review only

Qualitative data analysis

All qualitative data will be transcribed and entered into the analysis support software NVivo to support data management and analysis. Documentary sources will be added to the NVivo project file as PDF files. Visual approaches will be used to support the discussion and analysis of the pathways. Recordings of discussions will be analysed using a structured approach to conversation analysis. Interviews with women, partners and health professionals will be transcribed and analysed using a thematic framework approach, based on frameworks developed in recent work by the study team as part of the PROBIT-F trial (8, 26).

RESEARCH ETHICS APPROVAL AND DISSEMINATION

CHOICE has National Research Ethics Service Committee approval (York and Humber - Sheffield Research Ethics Committee, REC reference: 20/YH/0145), National R&D approval in Scotland (NHS Research Scotland Permissions) and England (Health Research Authority), and approval from the Public Benefit and Privacy Panel (PBPP) in Scotland is pending. The study will be conducted in accordance with the principles of the International Conference on Harmonisation Tripartite Guideline for Good Clinical Practice (ICH GCP).

CHOICE is registered on the ISRCTN registry (ISRCTN32652461)

Results will be submitted for peer reviewed academic publication and presented at international conferences. Meta-data produced in this study will also become available to Health Data Research UK (HDRUK) Gateway. Strengthening the Reporting of Observational Studies in Epidemiology (STROBE) guidance(16) and Reporting of studies Conducted using Observational Routinely-collected Data (RECORD) guidance(15) will be used to guide transparent reporting.

For peer review only

AUTHOR CONTRIBUTIONS

SJS, GCS, JS and DP contributed to the conception of the study. SJS, AB, MB, HC, CMc, KB, JS, NH, JH, FD, DP, NM, GCS and JN acquired funding. SJS, AB, MB, HC, CMc, KB, JS, NH, JH, FD, DP, NM, GCS, HR, CY, MH, MR, FW, DR and JN contributed to the study design. SJS, HR, AB and FW drafted the first version of the manuscript. SJS, AB, HR, MB, HC, CMc, KB, JS, NH, JH, FD, DP, NM, GCS, CY, MH, MR, FW, DR and JN revised the manuscript for important intellectual content and gave final approval of the version to be published.

PATIENT AND PUBLIC INVOLVEMENT

There are two PPI co-applicants on the study grant who have contributed to the protocol, study design and patient facing materials. A CHOICE Parent Advisory Group (PAG) has been established to provide further support and guidance on study materials and management.

ACKNOWLEDGMENTS

We gratefully acknowledge the help of the following people:

Trial Steering Committee: Dr Brenda Kelly (Chair), Dr Sarah Stock, Fiona Fraser, Jaqueline Birks, Dr Tracey Mills, Dr Kate Walker, Fiona Wee.

The West Midlands NIHR Clinical Research Network, who are the lead network for the study in England, and are providing support with site feasibility and set up.

Clevermed Ltd, provider of BadgerNet maternity records system, who are providing technical expertise and facilitating data extracts from approved sites.

For peer review only

The Edinburgh Clinical Trials Unit (ECTU), who are providing trial management, data management and statistical support.

Delivered Social, who kindly created and donated the CHOICE study website completely free of charge as part of their social impact programme; Delivered Social Green.

FUNDING

CHOICE is funded by the National Institute of Healthcare Research Health Technology and Assessment (NIHR HTA) NIHR 127569. The views expressed are those of the authors and not necessarily those of the NIHR or the Department of Health and Social Care.

SJS is supported by Wellcome Trust (209560/Z/17/Z)

COMPETING INTERESTS

SS reports grants from NIHR, grants from Wellcome Trust, grants from Tommy's Charity, grants from MRC, during the conduct of the study.

AB reports a grant from UK National Institute of Health Research, Research for patient benefit program (NIHR RfPB) for Trans-cervical balloon catheter and its comparison to sustained release prostaglandin use for out -patient induction of labour in low-risk women: A feasibility study for a randomised controlled trial. (PB-PG-0815-20022), outside the submitted work.

MR is an employee of Clevermed Ltd.

KB reports a grant from the NIHR during conduct of study

JS reports grants from NIHR during conduct of the study

NM leads and directs NNRD

For peer review only

GCS reports grants from NIHR HTA, during the conduct of the study; personal fees from GlaxoSmithKline Research and Development Limited, grants from Sera Prognostics Inc, non-financial support from Illumina Inc, grants, personal fees and non-financial support from Roche Diagnostics Ltd, grants from Medical Research Council, grants from NIHR HTA, grants from Wellbeing of Women, grants from Wellcome Trust, grants from NIHR, outside the submitted work; In addition, Dr. Smith has a patent Named inventor on patent application for a novel predictive test for fetal growth restriction (FGR) pending.

JN reports grants from University of Edinburgh, during the conduct of the study; and JN declares membership of the following NIHR boards: CPR Decision Making Committee, HTA Commissioning Board, HTA Commissioning Sub-Board (EOI), HTA Funding Boards Policy Group, HTA General Board, HTA Post-Board funding teleconference, NIHR CTU Standing Advisory Committee, NIHR HTA and EME Editorial Board and Pre-exposure Prophylaxis Impact Review Panel; and the MRC/NIHR EME Funding Board.

DATA STATEMENT

Individual NHS organisations are the data controllers for the study. We will not be able to share data for the study as we are not the data controllers. All results put into the public domain will be subject to statistical disclosure control according to usual processes. Meta-data produced in this study will be made available to Health Data Research UK (HDRUK) Gateway. Applications to use the Scottish datasets included in the study can be made via https://www.informationgovernance.scot.nhs.uk/pbpphsc/

For peer review only

REFERENCES

1. National Maternity and Perinatal Audit: Clinical Report 2019. Based on births in NHS maternity services between 1 April 2016 and 31 March 2017. London: RCOG; 2019.

2. Grobman WA, Rice MM, Reddy UM, et al. Labor Induction versus Expectant Management in Low-Risk Nulliparous Women. N Engl J Med 2018;379(6):513-23;

3. Stock SJ, Ferguson E, Duffy A, et al. Outcomes of elective induction of labour compared with expectant management: population based study. BMJ. 2012;344:e2838.

4. Knight HE, Cromwell DA, Gurol-Urganci I, et al. Perinatal mortality associated with induction of labour versus expectant management in nulliparous women aged 35 years or over: An English national cohort study. PLoS Med. 2017;14(11):e1002425.

5. Royal College of Midwives. The Gathering Storm: England's Midwifery Workforce Challenges, 2017

6. Shetty A, Burt R, Rice P, et al. Women's perceptions, expectations and satisfaction with induced labour--a questionnaire-based study. Eur J Obstet Gynecol Reprod Biol. 2005;123(1):56-61.

7. Henderson J, Redshaw M. Women's experience of induction of labor: a mixed methods study. Acta Obstet Gynecol Scand. 2013;92(10):1159-67.

8. Coates R, Cupples G, Scamell A, et al. Women's experiences of induction of labour: qualitative systematic review and thematic synthesis. Midwifery. 2018.

For peer review only

9. Norman JE, Stock S. Intracervical Foley catheter for induction of labour. Lancet. 2011;378(9809):2054-5.

10. NICE Guideline CG70. Induction of labour 2008.

11. Jozwiak M, Oude Rengerink K, Benthem M, et al. Foley catheter versus vaginal prostaglandin E2 gel for induction of labour at term (PROBAAT trial): an open-label, randomised controlled trial. Lancet. 2011;378(9809):2095-103.

12. Alfirevic Z, Gyte GML, Nogueira Pileggi V, et al. Home versus in patient induction of labour for improving birth outcomes. Cochrane Database of Systemic Reviews 2020, Issue8. Art No.: CD007372.DOI:10.1002/14651858.CD007372.pub4

13. Cheyne H, McCourt C, Semple K. Mother knows best: developing a consumer led, evidence informed, research agenda for maternity care. Midwifery. 2013;29(6):705-12

14. Dos Santos F, Drymiotou S, Antequera A, et al. Development of a core outcome set for trials on induction of labour (COSIOL): An international multi-stakeholder Delphi study. BJOG. 2018.

15. Benchimol EI, Smeeth L, Guttmann A, et al; RECORD Working Committee. The REporting of studies Conducted using Observational Routinely-collected health Data (RECORD) statement. PLoS Med. 2015 Oct 6;12(10):e1001885. doi: 10.1371/journal.pmed.1001885. eCollection 2015 Oct.

16. von Elm E, Altman DG, Egger M, et al; STROBE Initiative. The Strengthening the Reporting of Observational Studies in Epidemiology (STROBE) statement: guidelines for reporting observational studies. PLoS Med. 2007 Oct 16;4(10):e296.

For peer review only

17. Jozwiak M, Oude Rengerink K, Ten Eikelder ML, et al. Foley catheter or prostaglandin E2 inserts for induction of labour at term: an open-label randomized controlled trial (PROBAAT-P trial) and systematic review of literature. Eur J Obstet Gynecol Reprod Biol. 2013;170(1):137-45.

18. Consolidated Health Economic Evaluation Reporting Standards (CHEERS) statement BMJ 2013; 346 doi: https://doi.org/10.1136/bmj.f1049

19. Unit Costs of Health and Social Care 2018: University of Kent at Canterbury: PSSRU, 2018

20. Kreif N, Grieve R, Sadique MZ. Statistical methods for cost-effectiveness analyses that use observational data: a critical appraisal tool and review of current practice. Health Econ. 2013;22(4):486-500

21. https://www.onlinesurveys.ac.uk
Accessed December 2020

22. Vogel JP, Osoti AO, Kelly AJ, et al. Pharmacological and mechanical interventions for labour induction in outpatient settings. Cochrane Database Syst Rev. 2017;9:CD007701.

23. Adelson PL, Wedlock GR, Wilkinson CS, et al. A cost analysis of inpatient compared with outpatient prostaglandin E2 cervical priming for induction of labour: results from the OPRA trial. Aust Health Rev. 2013;37(4):467-73.

24. Sharp AN, Stock SJ, Alfirevic Z. Outpatient induction of labour in the UK: a survey of practice. Eur J Obstet Gynecol Reprod Biol. 2016;204:21-3.

For peer review only

25. Kelly AJ, Alfirevic Z, Ghosh A. Outpatient versus inpatient induction of labour for improving birth outcomes. Cochrane Database Syst Rev. 2013(11):CD007372.

26. Rose Coates, Georgina Cupples, Amanda Scamell, et al. Women's experiences of outpatient induction of labour with double balloon catheter or prostaglandin pessary: A qualitative study, Women and Birth (2020), https://doi.org/10.1016/j.wombi.2020.07.00

For peer review only

For peer review only

FIGURE LEGEND

Figure 1: Logic model for the qCHOICE process evaluation

For peer review only

TABLES

Table 1: Secondary outcomes

Safety outcomes	*Baby*	*Maternal*
	Any neonatal unit admission (any level of care)	Intensive care unit transfer
	Neonatal intensive care unit (NICU) admission	High dependency level care
	Duration of neonatal unit stay	Hyperstimulation or tachysystole (as defined by care givers)
	Duration of NICU stay	Hyperstimulation or tachysystole causing CTG abnormality (as defined by care givers)
	APGAR score <7 at 5 minutes	Umbilical cord prolapse
	APGAR score <4 at 5 minutes	Birth outwith hospital
	Arterial Cord Blood pH <7.1	Postpartum haemorrhage 1000ml or more
	Arterial Cord base excess >12mmol/L	Maternal pyrexia 38 °C or more after commencing cervical ripening (*Exploratory outcome)*
	Neonatal Seizures	
	Hypoxic Ischaemic Encephalopathy (as recorded by care givers)	
	Level 2 or Level 3 Hypoxic Ischaemic Encephalopathy (as recorded by care givers)	

For peer review only

	Meconium aspiration syndrome<br><br>Mechanical ventilation<br><br>Intracranial haemorrhage<br><br>Stillbirth after admission/first attendance for induction of labour (excluding deaths from congenital anomalies)<br><br>Early neonatal death up to 7 days after birth (day 0-6; excluding deaths from congenital anomalies)<br><br>Treatment for neonatal sepsis [defined as positive blood, cerebral spinal fluid, or urine culture or cardiovascular collapse or X-ray confirming infection] *(Exploratory outcome)*<br><br>Treatment in neonatal unit for neonatal infection (defined as antibiotic treatment and Temperature ≥37.5 °C or <35.5 °C) *(Exploratory outcome)*<br><br>Treatment for neonatal jaundice [defined as peak total bilirubin of at least 15mg or the use of phototherapy] *(Exploratory outcome)*	
**Effectiveness outcomes**	Time from first cervical ripening agent to admission to labour ward/birth unit<br><br>Time from first cervical ripening agent to birth	

For peer review only

	More than one cervical ripening agent used<br><br>Duration of antenatal hospital stay for cervical ripening<br><br>Duration of labour ward admission until birth<br><br>Duration postnatal hospital stay (mother)<br><br>Total hospital stay<br><br>Hours spent at home<br><br>Oxytocin use<br><br>Mode of birth<br><br>Birth in obstetric unit<br><br>Birth in alongside midwifery unit (if available at that site)
**Mother baby outcomes**	Breastfeeding at discharge from maternity care<br><br>Skin to skin at birth
**Cost effectiveness**	***Primary outcomes***<br><br>Incremental cost per neonatal admissions avoided (home versus in-hospital)<br><br>Incremental quality adjusted life year (QALYs) (home versus in-hospital)

For peer review only

	Other (exploratory) economic outcomes<br><br>Incremental cost per hour prevented from hospital admission to delivery/birth<br><br>Incremental cost per neonatal admission avoided (home balloon catheter versus home dinoprostone)<br><br>Incremental cost per QALY (home balloon catheter versus home dinoprostone)<br><br>Incremental cost per hour prevented from hospital admission to delivery/birth (home balloon catheter versus home dinoprostone
**Outcomes to check comparability of groups/matching**	Birthweight<br><br>Birthweight centile<br><br>Small for gestational age (<10th centile for gestational age)<br><br>Large for gestational age (>90th centile for gestational age)

For peer review only

**qCHOICE process evaluation outcomes**	***Primary Outcome***<br><br>Sense of control (agentry) in labour<br><br>***Secondary Outcomes***<br><br>Women's satisfaction with IOL care<br><br>Women's postnatal psychological wellbeing<br><br>Women's overall evaluation of their labour and birth experience (qualitative analysis)<br><br>Costs incurred by the woman and family

For peer review only

Figure 1. Process evaluation logic model – V1 18/12/18

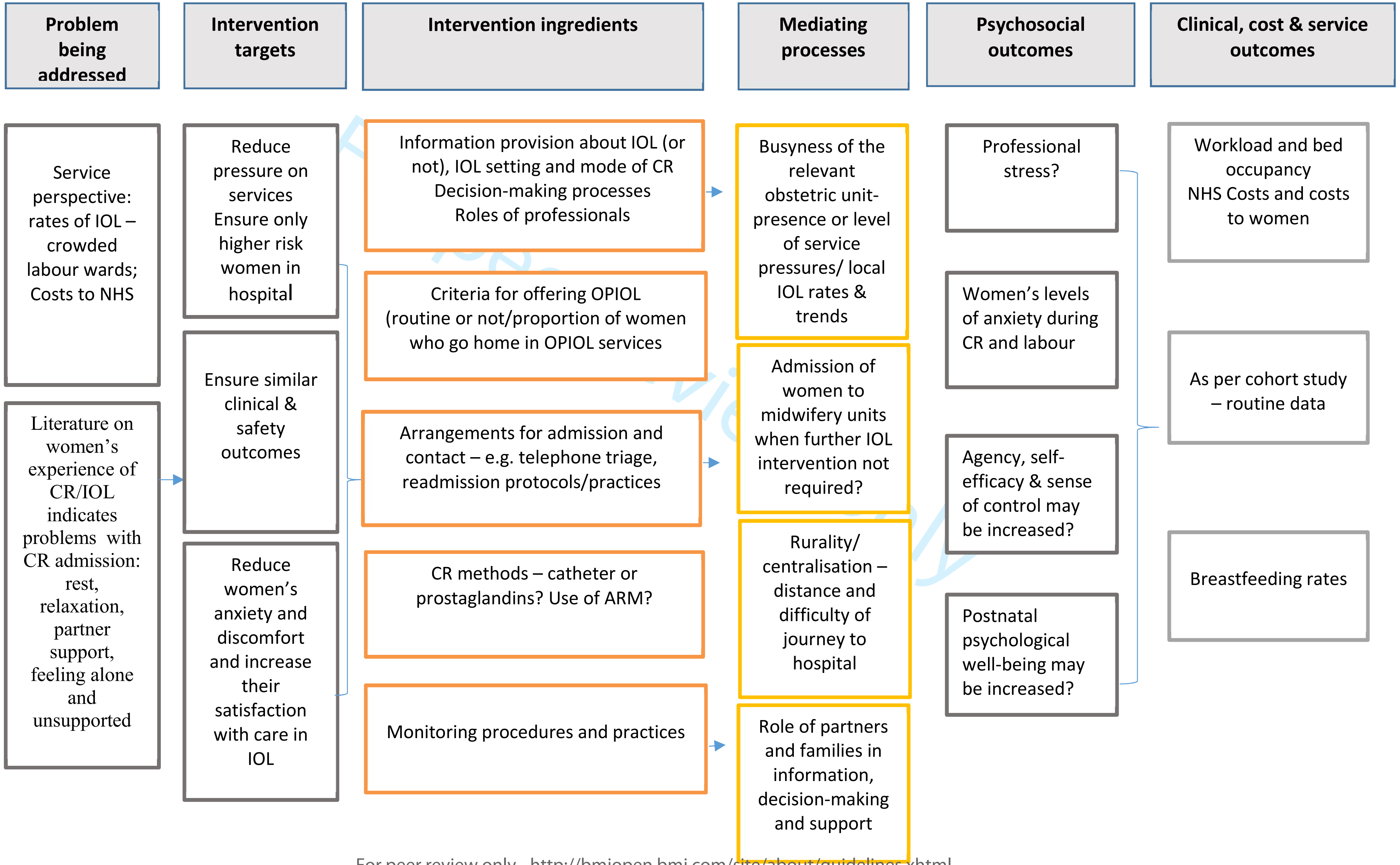

Supplement: Author's manuscript [file bmjopen-2021-050452.draft_revisions.pdf]
