# Supplementary material for: Cervical ripening at home or in-hospital—prospective cohort study and process evaluation (CHOICE) study: a protocol
Source: BMJ Open. 2021 May 4;11(5):e050452. doi: 10.1136/bmjopen-2021-050452 (PMC8098973; doi:10.1136/bmjopen-2021-050452)
Supplement: Reviewer comments [file bmjopen-2021-050452.reviewer_comments.pdf]

PEER REVIEW HISTORY

BMJ Open publishes all reviews undertaken for accepted manuscripts. Reviewers are asked to complete a checklist review form (**http://bmjopen.bmj.com/site/about/resources/checklist.pdf**) and are provided with free text boxes to elaborate on their assessment. These free text comments are reproduced below.

ARTICLE DETAILS

**TITLE (PROVISIONAL)**	Cervical ripening at home or in-hospital - prospective cohort study and process evaluation (CHOICE) study: Protocol
**AUTHORS**	Stock, Sarah; Bhide, Amarnath; Richardson, Heather; Black, Mairead; Yuill, Cassandra; Harkness, Mairi; Reid, Maggie; Wee, Fiona; Cheyne, Helen; McCourt, Christine; Rana, Dikshyanta; Boyd, Kathleen; Sanders, Julia; Heera, Neelam; Huddleston, Jane; Denison, Fiona; Pasupathy, Dharmintra; Modi, Neena; Smith, Gordon; Norrie, John

This article was not externally reviewed at BMJ Open. Protocols that have been independently assessed prior to submission to BMJ Open are usually fast-tracked to publication on the grounds that further substantial changes will not be possible. This independent assessment will usually be external, independent review for both a substantial grant award from a non-commercial or government funder and ethics approval.
